# Real-world effectiveness of adding newer generation GLP-1RA to SGLT2i in type 2 diabetes

**DOI:** 10.1186/s12933-025-02737-1

**Published:** 2025-04-24

**Authors:** Nathorn Chaiyakunapruk, Xi Tan, Yuanjie Liang, Mico Guevarra, Lin Xie, Alice Y. Y. Cheng

**Affiliations:** 1 Department of Pharmacotherapy, University of Utah College of Pharmacy, Salt Lake City, UT USA; 2 IDEAS Center, Veterans Affairs Salt Lake City Healthcare System, Salt Lake City, UT USA; 3 Novo Nordisk Inc, Plainsboro, NJ USA; 4 Department of Medicine, University of Toronto, 507-2300 Eglinton Avenue West, Mississauga, ON L5M 2V8 Canada

**Keywords:** Sodium-glucose cotransporter 2 inhibitor, Glucagonlike peptide-1 receptor agonist, Type 2 diabetes, Atherosclerotic cardiovascular disease, Chronic kidney disease, Major adverse cardiovascular event, Combination therapy

## Abstract

**Background:**

Guidelines recommend combination therapy with glucagonlike peptide-1 receptor agonists (GLP-1RAs) and sodium-glucose cotransporter 2 inhibitors (SGLT2is) for cardiorenal risk reduction in people with type 2 diabetes (T2D); however, there is limited real-world evidence on the long-term effects of combination therapy on cardiometabolic and renal outcomes. The objective of this study was to assess cardiovascular (CV), metabolic, and renal effects of combination therapy with newer generation GLP-1RA (including once-weekly GLP-1RAs, once-daily oral semaglutide, and dual GLP-1/glucose-dependent insulinotropic polypeptide [GIP] agonists) and SGLT2i compared with SGLT2i alone.

**Methods:**

This retrospective cohort study included data on US adults with T2D receiving SGLT2i from Komodo’s Healthcare Map from January 1, 2017, to June 30, 2023. The study included 100,455 people in the combination GLP-1RA and SGLT2i group and 339,540 people in the comparison SGLT2i group across 3 cohorts: T2D with atherosclerotic cardiovascular disease (ASCVD), T2D, and T2D with chronic kidney disease (CKD). Entropy balancing was used to balance patient characteristics. Time to first event of ischemic stroke, myocardial infarction (MI), 3-point major adverse cardiovascular event (MACE), and 5-point MACE in T2D with ASCVD cohort were measured. In the T2D cohort, follow-up and change in glycated hemoglobin (HbA_1c_) and weight, odds of achieving HbA_1c_ < 7% and HbA_1c_ < 8%, and odds of achieving 5%, 10%, and 15% decrease in weight were also measured.

**Results:**

The combination and comparison groups included 34,690 and 130,220 people, respectively, in the T2D with ASCVD cohort; 8,220 and 22,891 people, respectively, in the T2D cohort; and 8,783 and 35,532 people, respectively, in the T2D with CKD cohort. Compared with SGLT2i alone, combination therapy was significantly associated with 42% lower risk of ischemic stroke, 37% lower risk of MI, 46% lower risk of 3-point MACE, and 45% lower risk of 5-point MACE among people with T2D and ASCVD. Among the individual GLP-1RAs assessed, the largest reductions in CV risk, HbA_1c_, and weight outcomes were observed with combination therapy with SGLT2i and once weekly semaglutide for T2D.

**Conclusions:**

Combination of SGLT2i and GLP-1RA achieved significantly better cardiometabolic outcomes compared with SGLT2i alone; this supports the hypothesis that the cardioprotective benefits of GLP-1RA and SGLT2i may be additive.

**Graphical abstract:**

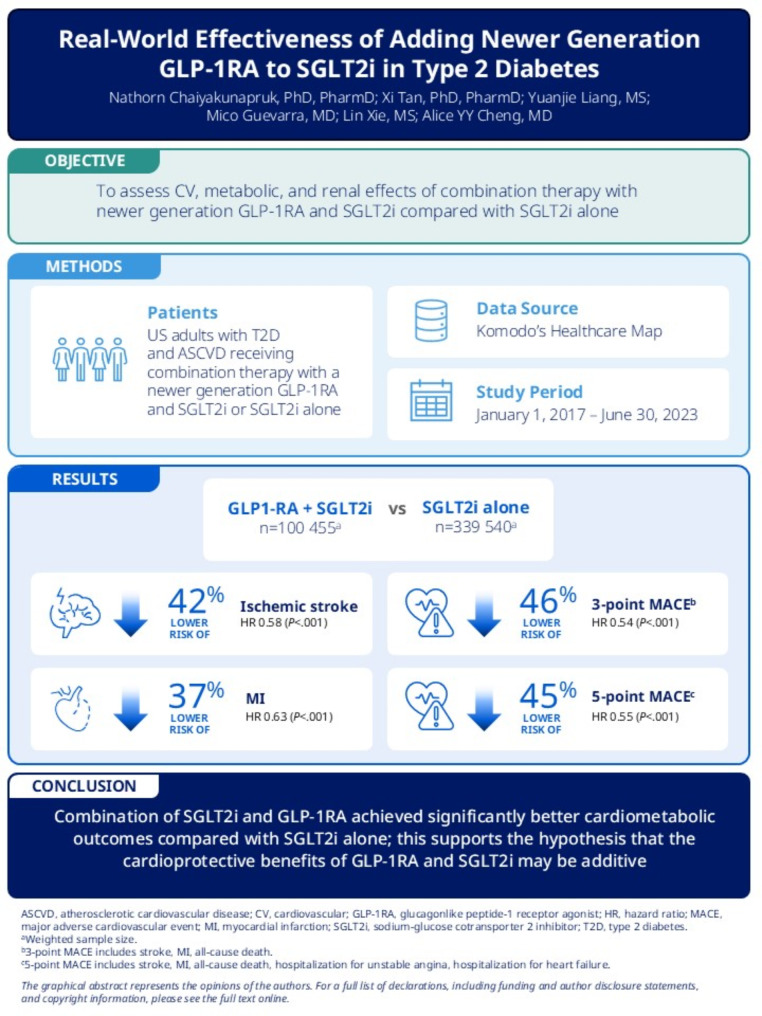

**Supplementary Information:**

The online version contains supplementary material available at 10.1186/s12933-025-02737-1.

## Research insights

**What is currently known about this topic?**

- Guidelines advise therapy with GLP-1RA and SGLT2i for cardiorenal risk reduction in people with T2D.
- Real-world data on long-term effects of combination therapy on cardiometabolic outcomes are limited.

**What is the key research question?**

- What are the additive cardiometabolic effects of combination therapy with newer generation GLP-1RA and SGLT2i vs SGLT2i alone among US adults with T2D and ASCVD?

**What is new?**

- Combination therapy was associated with lower risk of myocardial infarction vs SGLT2i therapy alone.
- Combination therapy was associated with lower risk of stroke vs SGLT2i therapy alone.
- Combination therapy was associated with lower risk of 3- and 5-point MACE vs SGLT2i therapy alone.

**How might this influence clinical practice?**

- Combination therapy of GLP-1RA and SGLT2i could lead to better clinical outcomes.

## Background

Type 2 diabetes (T2D) is a progressive disease associated with increased risk of complications, including atherosclerotic cardiovascular disease (ASCVD) and chronic kidney disease (CKD) [1]. ASCVD is the leading cause of morbidity and mortality in people with T2D [2, 3]. CKD is estimated to develop in ~ 40–50% of people with T2D; the presence of CKD increases cardiovascular risk and economic burden [4, 5]. 

Guidelines recommend glucagonlike peptide-1 receptor agonist (GLP-1RA) and/or sodium-glucose cotransporter 2 inhibitor (SGLT2i) as initial pharmacologic therapy in people with T2D and ASCVD or high risk for ASCVD, heart failure, or CKD for comprehensive cardiovascular (CV) and renal risk management; combination therapy with SGLT2i and GLP-1RA is recommended for this same population when glycemic targets have not been met with monotherapy [6, 7]. In the United States, use of GLP-1RA with SGLT2i in guideline-recommended target populations with T2D has increased in recent years; however, adoption of combination therapies remains suboptimal [8, 9]. Clinical trials and real-world studies have shown additive benefits with the combination of SGLT2i and GLP-1RA for glycemic and weight control in T2D [10–16]. In addition, GLP-1RA and SGLT2i have individually demonstrated CV and kidney risk reductions in people with T2D [17–21]. 

Despite the guideline-recommended use of both SGLT2i and GLP-1RA therapy, there is a paucity of real-world evidence on the long-term effects of combining SGLT2is with newer generation GLP-1RAs (including once-weekly GLP-1RAs, once-daily oral semaglutide, and dual GLP-1/glucose-dependent insulinotropic polypeptide [GIP] agonists) on metabolic outcomes and other cardiorenal outcomes [22]. The aim of this study was to assess additive CV, metabolic, and renal effects of combination therapy with SGLT2i and newer generation GLP-1RA compared with SGLT2i alone among people with T2D with ASCVD, T2D, and T2D with CKD, respectively.

## Methods

### Study design

This retrospective, observational cohort study used data from Komodo’s Healthcare Map to compare outcomes between people using both newer generation GLP-1RA and SGLT2i and those using SGLT2i without GLP-1RA (See Supplementary Fig. 1, Supplementary Materials). This study adhered to the Strengthening the Reporting of Observational Studies in Epidemiology reporting guidelines [23]. The study period was from July 1, 2017, to June 30, 2023.

The combination group included those who added a newer generation GLP-1RA (i.e., exenatide once weekly [OW], dulaglutide, semaglutide OW T2D, or oral semaglutide) or dual GLP-1/GIP agonist (tirzepatide T2D) to their SGLT2i (i.e., canagliflozin, dapagliflozin, empagliflozin, ertugliflozin, or bexagliflozin) treatment, with persistent use of a GLP-1RA and SGLT2i for ≥ 180 days each and an overlap ≥ 120 days, where the gap after exhaustion of days’ supply was required to be ≤ 30 days. Switching within SGLT2i or newer generation GLP-1RA class was allowed. The index date for the combination group was defined as the date of the first prescription of newer generation GLP-1RA. The comparison group included those who used SGLT2i and did not use any GLP-1RA; the index date for the comparison group was randomly chosen among weighted dates of SGLT2i prescriptions for each individual to match the distribution of time from the first observed SGLT2i prescription to the index date in the combination group. People in the combination and comparison groups were followed for at least 6 months (180 days) after the index date and until the earliest event occurrence or censoring, including discontinuation of either SGLT2i or GLP-1RA, switch to or addition of an older generation GLP-1RA, lapse in continuous enrollment, or the end of the study period.

### Data source

Komodo’s Healthcare Map contains longitudinal anonymized patient-level US pharmacy and medical claims data on over 330 million patient journeys. Patient encounter data in Komodo’s Healthcare Map are derived directly from payer sources, including fully integrated fee-for-service Medicare data, Medicare Advantage claims, commercial claims, and Medicaid claims. Data are representative of the US census population in terms of geographical, sex, and age distribution.

### Study population

The study population included US adults with confirmed T2D treated with SGLT2i. To maximize sample sizes whilst aligning with guideline-recommended target populations for these therapies, different whose patient characteristics were independently well-matched: (1) CV outcomes among people with T2D and ASCVD, (2) glucose and weight outcomes among people with T2D, and (3) renal outcomes in people with T2D and CKD [6]. 

For all cohorts, patients were required to be ≥ 18 years of age on January 1, 2017 with a confirmed T2D diagnosis (≥ 2 claims for *International Classification of Diseases*,* Tenth Revision*,* Clinical Modification* [ICD-10-CM] E11 and subcodes on distinct days in the study period), ≥ 1 T2D diagnosis in the baseline period, ≥ 2 prescription claims for SGLT2i in the study period, ≥ 180 days persistent use of the index drug(s), and continuous enrollment for the baseline and follow-up periods (See Supplementary Fig. 2, Supplementary Materials). Patients in the combination group were required to have initiated a newer generation GLP-1RA after the first prescription of SGLT2i and ≥ 120 days’ supply of SGLT2i and newer generation GLP-1RA. Patients meeting the following criteria were excluded from the analysis: evidence of type 1 diabetes or pregnancy in the study period, missing age or sex data, initiation of another new glucose-lowering therapy on the index date, and use of any GLP-1RA in the baseline period.

In the T2D cohort, patients in the combination and comparison groups were required to have valid baseline and follow-up glycated hemoglobin (HbA_1c_) and weight measures available to be included. Patients who had bariatric surgery or used obesity management medications during the study period were excluded.

In the T2D with ASCVD cohort, patients in the combination and comparison groups were required to have a history of ASCVD and were excluded if they had a major adverse cardiovascular event outcome within 60 days before the index date. History of ASCVD was defined by ICD-10-CM codes I63 (excluding I63.1, I63.4, and I63.6), G45, I65, I66, I67.2, I67.81, I67.82, I69 (excluding I69.0, I69.1, and I69.2), I21, I22, I23, I24, I20, I25 (excluding 125.3, and I25.4), I70, I73.9, I74, and I75 [24]. 

In the T2D with CKD cohort, patients in the combination and comparison groups were required to have stage 2, 3, 4, or 5 CKD (excluding end-stage kidney disease) at baseline. CKD stages 2–5 were identified using ICD-10-CM codes (N18.2, N18.3, N18.4, N18.5) or evidence of 2 estimated glomerular filtration rates (eGFRs) ≤ 89 mL/min/1.73m^2^; patients were additionally required to have valid eGFR records during baseline and follow-up.

### Primary outcomes

CV outcomes were assessed for those in the T2D with ASCVD cohort. Time to first ischemic stroke event in the follow-up was identified as a primary diagnosis of inpatient claims using the ICD-10-CM code I63 (except I63.1, I63.4, I63.6). One inpatient visit for stroke was considered 1 stroke event. Time to first myocardial infarction (MI) event in the follow-up was identified as a primary diagnosis in the inpatient setting using ICD-10-CM codes I21 and I22. One inpatient visit for MI was considered 1 MI event. Time to 3- and 5-point major adverse cardiovascular event (MACE) in the follow-up was also measured [25]. Three-point MACE included nonfatal ischemic stroke, nonfatal MI, and all-cause death. Five-point MACE included nonfatal ischemic stroke, nonfatal MI, all-cause death, hospitalization for unstable angina (primary diagnosis in the inpatient setting using ICD-10-CM code I20.0), and hospitalization for heart failure (primary diagnosis in the inpatient setting using ICD-10-CM code I50).

HbA_1c_ and weight outcomes were assessed for those in the T2D cohort. Baseline HbA_1c_, weight, and body mass index (BMI) were measured at days − 120 and 0 (day 0 = index date), and follow-up HbA_1c_ was measured at 180 days (day 179 ± 60 days), 360 days (day 359 ± 60 days), and 540 days (day 539 ± 60 days) after index. The HbA_1c_, weight, and BMI values closest to the index date were used if multiple measures were available during the baseline period. Values closest to the 180-, 360-, and 540-day time points were used if multiple follow-up values were available. The change in HbA_1c_ from baseline to each of the 180-, 360-, and 540-day time points was calculated. The proportions of people achieving HbA_1c_ < 7% and < 8% during the follow-up period were assessed. Changes in weight and BMI from baseline to each of the 180-, 360-, and 540-day time points were calculated. The proportions of people with a 5%, 10%, and 15% decrease in weight during the follow-up period were reported.

### Secondary outcomes

Renal outcomes were assessed for those in the T2D with CKD cohort. Change in eGFR from baseline to each of the 180-, 360-, and 540-day time points was calculated.

### Individual index drug comparisons

Primary and secondary outcomes were also compared between people treated with individual index newer generation GLP-1RA in combination with SGLT2i and those treated with SGLT2i alone. Newer generation GLP-1RAs included in individual drug level comparisons included semaglutide OW T2D, oral semaglutide, and dulaglutide; exenatide OW and tirzepatide T2D were not included in individual drug level comparisons due to limited sample sizes.

### Statistical analyses

Descriptive analyses were performed for all outcomes. Counts and frequencies were used for categorical variables; means and SDs were used for continuous variables. Incidence rates, defined as the number of events divided by the follow-up time, were reported per 1000 person-years (PY) for CV outcomes. Entropy balancing was applied to bivariate and multivariate analyses to compare outcomes between the combination and comparison groups [26, 27]. The target variables for entropy balancing can be found in Supplementary Table 1 (See Supplementary Materials). Average treatment effect (ATE) was used for drug class–level comparisons (SGLT2i + GLP-1RA vs. SGLT2i), and average treatment effect of the treated (ATT) was used for individual GLP-1RA drug comparisons (SGLT2i + individual GLP-1RA vs. SGLT2i) as the ATE target was not feasible.

Weighted and unweighted descriptive statistics were reported for all covariates. Standardized mean differences (SMDs) were presented, and SMD ≥ 0.1 was considered a meaningful difference [28–30]. 

Generalized linear models using appropriate distribution and function were used. For time-to-event outcomes, weighted Cox proportional hazards regressions were conducted. Sensitivity analyses stratified by age group (individuals ≥ 65 years of age and individuals < 65 years of age) were also performed.

## Results

### Baseline characteristics

After applying inclusion and exclusion criteria, the T2D with ASCVD cohort included 34,960 and 130,220 people in the combination and comparison groups, respectively (See Supplementary Fig. 2, Supplementary Materials). The T2D cohort included 8220 and 22,891 people in the combination and comparison groups, respectively. The T2D with CKD cohort included 8783 and 35,532 people in the combination and comparison groups, respectively.

After weighting, no significant differences in baseline characteristics were observed between the groups in all cohorts. The mean age was 64, 61, and 67 years in the T2D with ASCVD cohort, T2D cohort, and T2D with CKD cohort, respectively. Detailed unweighted and weighted baseline characteristics are listed in Supplementary Tables 2–4 (See Supplementary Materials).

### Primary CV outcomes for T2D with ASCVD cohort

**Fig. 1:**
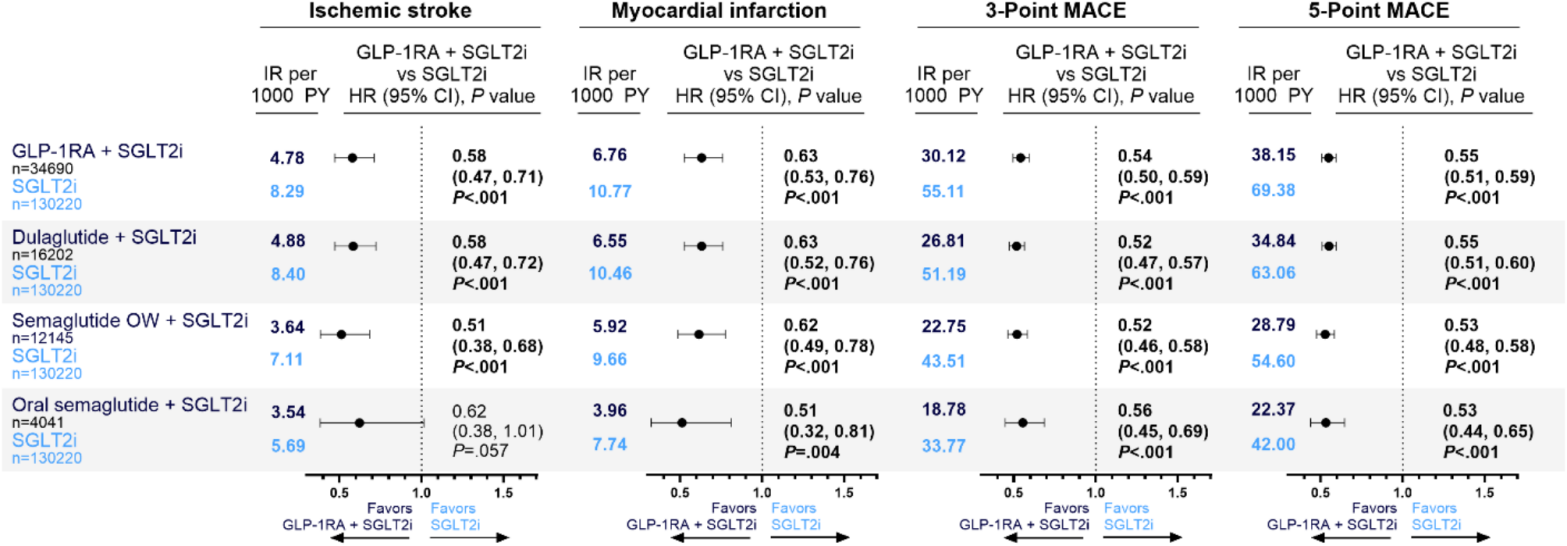
Weighted CV outcomes for GLP-1RA and SGLT2i compared with SGLT2i alone. Weighted IRs per 1000 PY of ischemic stroke, MI, 3-point MACE, and 5-point MACE among adults with T2D and ASCVD using combination GLP-1RA (including semaglutide OW T2D, oral semaglutide, dulaglutide, exenatide OW, and tirzepatide T2D) and SGLT2i therapy or SGLT2i therapy alone. Adjusted HRs for ischemic stroke, MI, 3-point MACE, and 5-point MACE among adults with T2D and ASCVD using combination GLP-1RA and SGLT2i therapy compared with SGLT2i alone. Bold HRs indicate statistical significance. ASCVD indicates atherosclerotic cardiovascular disease; CV, cardiovascular; GLP-1RA, glucagonlike peptide-1 receptor agonist; HR, hazard ratio; IR, incidence rate; MACE, major adverse cardiovascular event; MI, myocardial infarction; OW, once weekly; PY, person-years; SGLT2i, sodium-glucose cotransporter 2 inhibitor; T2D, type 2 diabetes

People in the combination group had lower incidence rates of ischemic stroke (4.78 per 1000 PY vs. 8.30 per 1000 PY), MI (6.76 per 1000 PY vs. 10.77 per 1000 PY), 3-point MACE (30.12 per 1000 PY vs. 55.12 per 1000 PY), and 5-point MACE (38.15 per 1000 PY vs. 69.38 per 1000 PY) than those in the comparison group (Fig. 1). Compared with SGLT2i alone, combination therapy was significantly associated with 42% lower risk of ischemic stroke (hazard ratio [HR], 0.58; 95% CI, 0.47–0.71; *P* <.001), 37% lower risk of MI (HR, 0.63; 95% CI, 0.53–0.76; *P* <.001), 46% lower risk of 3-point MACE (HR, 0.54; 95% CI, 0.50–0.59; *P* <.001), and 45% lower risk of 5-point MACE (HR, 0.55; 95% CI, 0.51–0.59; *P* <.001).

Compared with SGLT2i alone, combination of SGLT2i with semaglutide OW T2D was significantly associated with 49% lower risk of ischemic stroke (HR, 0.51; 95% CI, 0.38–0.68; *P* <.001), 38% lower risk of MI (HR, 0.62; 95% CI, 0.49–0.78; *P* <.001), 48% lower risk of 3-point MACE (HR, 0.52; 95% CI, 0.46–0.58; *P* <.001), and 47% lower risk of 5-point MACE (HR, 0.53; 95% CI, 0.48–0.58; *P* <.001). Compared with SGLT2i alone, combination therapy of SGLT2i with oral semaglutide was significantly associated with 49% lower risk of MI (HR, 0.51; 95% CI, 0.32–0.81; *P* =.004), 44% lower risk of 3-point MACE (HR, 0.56; 95% CI, 0.45–0.69; *P* <.001), and 47% lower risk of 5-point MACE (HR, 0.53; 95% CI, 0.44–0.65; *P* <.001). No significant difference in ischemic stroke was observed. Compared with SGLT2i alone, combination therapy of SGLT2i with dulaglutide was significantly associated with 42% lower risk of ischemic stroke (HR, 0.58; 95% CI, 0.47–0.72; *P* <.001), 37% lower risk of MI (HR, 0.63; 95% CI, 0.52–0.76; *P* <.001), 48% lower risk of 3-point MACE (HR, 0.52; 95% CI, 0.47–0.57; *P* <.001), and 45% lower risk of 5-point MACE (HR, 0.55; 95% CI, 0.51–0.60; *P* <.001).

### Primary HbA_1c_ outcomes for T2D cohort

**Fig. 2:**
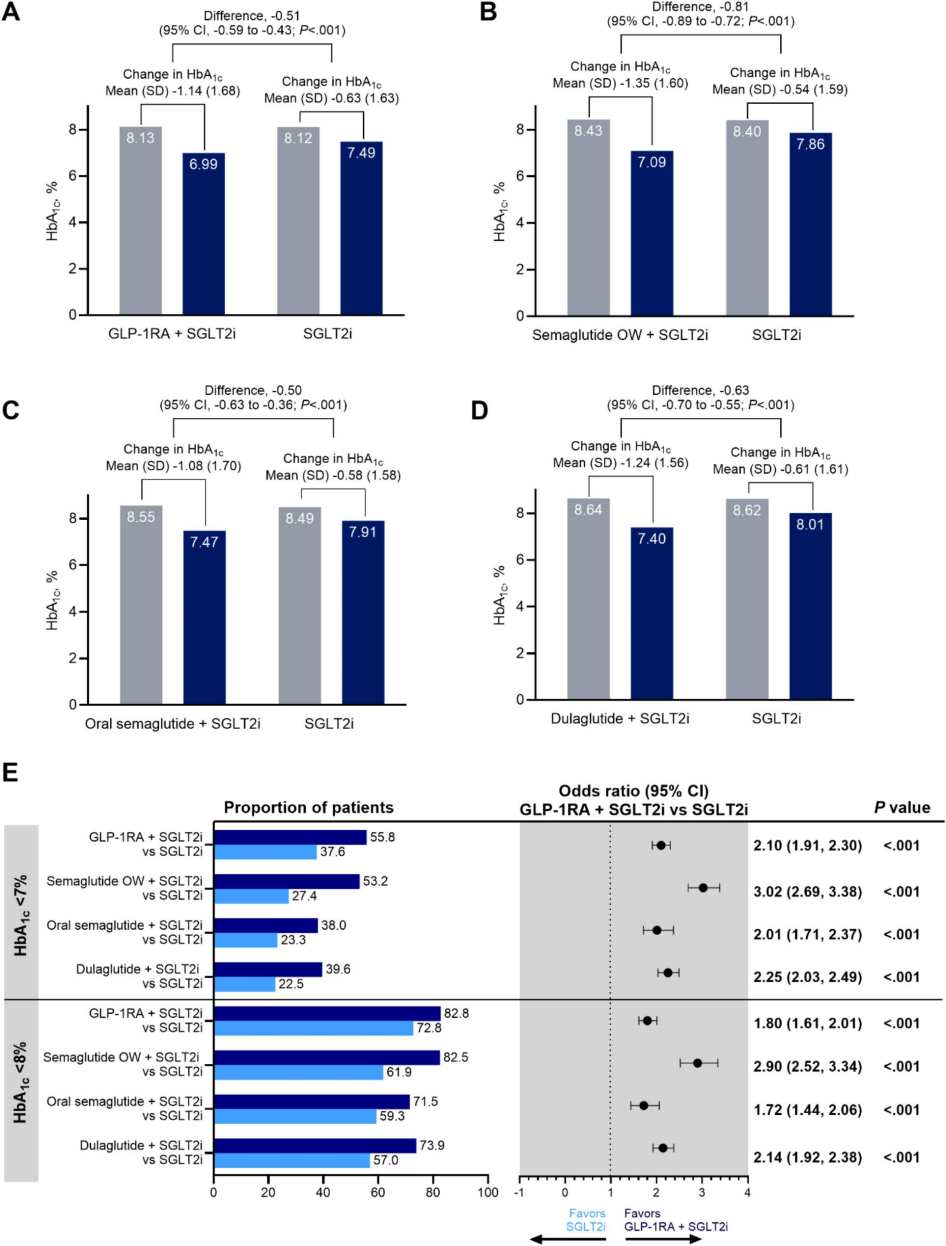
Weighted HbA_1c_ outcomes at 6 months for GLP-1RA and SGLT2i compared with SGLT2i alone. Weighted baseline (gray bars) and 6-month follow-up HbA_1c_ levels (dark blue bars) among adults with T2D using **A** combination of GLP-1RA (including semaglutide OW T2D, oral semaglutide, dulaglutide, exenatide OW, and tirzepatide T2D) and SGLT2i compared with SGLT2i alone, **B** combination of semaglutide OW and SGLT2i compared with SGLT2i alone, **C** combination of oral semaglutide and SGLT2i compared with SGLT2i alone, and **D** combination of dulaglutide and SGLT2i compared with SGLT2i alone. **E** Weighted descriptive statistics and odds ratios of achieving HbA_1c_ < 7% or HbA_1c_ < 8% among adults with T2D using combination GLP-1RA (including semaglutide OW T2D, oral semaglutide, dulaglutide, exenatide OW, and tirzepatide T2D) and SGLT2i therapy (dark blue bars) compared with SGLT2i alone (light blue bars). Bold odds ratios indicate statistical significance. GLP-1RA indicates glucagonlike peptide-1 receptor agonist; HbA_1c_, glycated hemoglobin; OW, once weekly; SGLT2i, sodium-glucose cotransporter 2 inhibitor; T2D, type 2 diabetes

Compared with SGLT2i at 6 months, combination therapy was significantly associated with 0.51% greater reduction in HbA_1c_ from baseline (Fig. 2). At 12 and 18 months, respectively, 0.44% and 0.46% greater reductions in HbA_1c_ from baseline were observed with combination therapy compared with SGLT2i (See Supplementary Figs. 3, 4, Supplementary Materials).

People in the combination SGLT2i with semaglutide OW T2D group had 0.81%, 0.74%, and 0.79% greater reductions in HbA_1c_ from baseline at 6 (Fig. 2), 12 (See Supplementary Fig. 3, Supplementary Materials), and 18 months (See Supplementary Fig. 4, Supplementary Materials), respectively, compared with the SGLT2i group. People in the combination SGLT2i with oral semaglutide group had 0.50%, 0.44%, and 0.53% greater reductions in HbA_1c_ from baseline at 6, 12, and 18 months, respectively. People in the combination SGLT2i with dulaglutide group had 0.63%, 0.52%, and 0.58% greater reductions in HbA_1c_ from baseline at 6, 12, and 18 months, respectively.

### Primary weight and BMI outcomes for T2D cohort

**Fig. 3:**
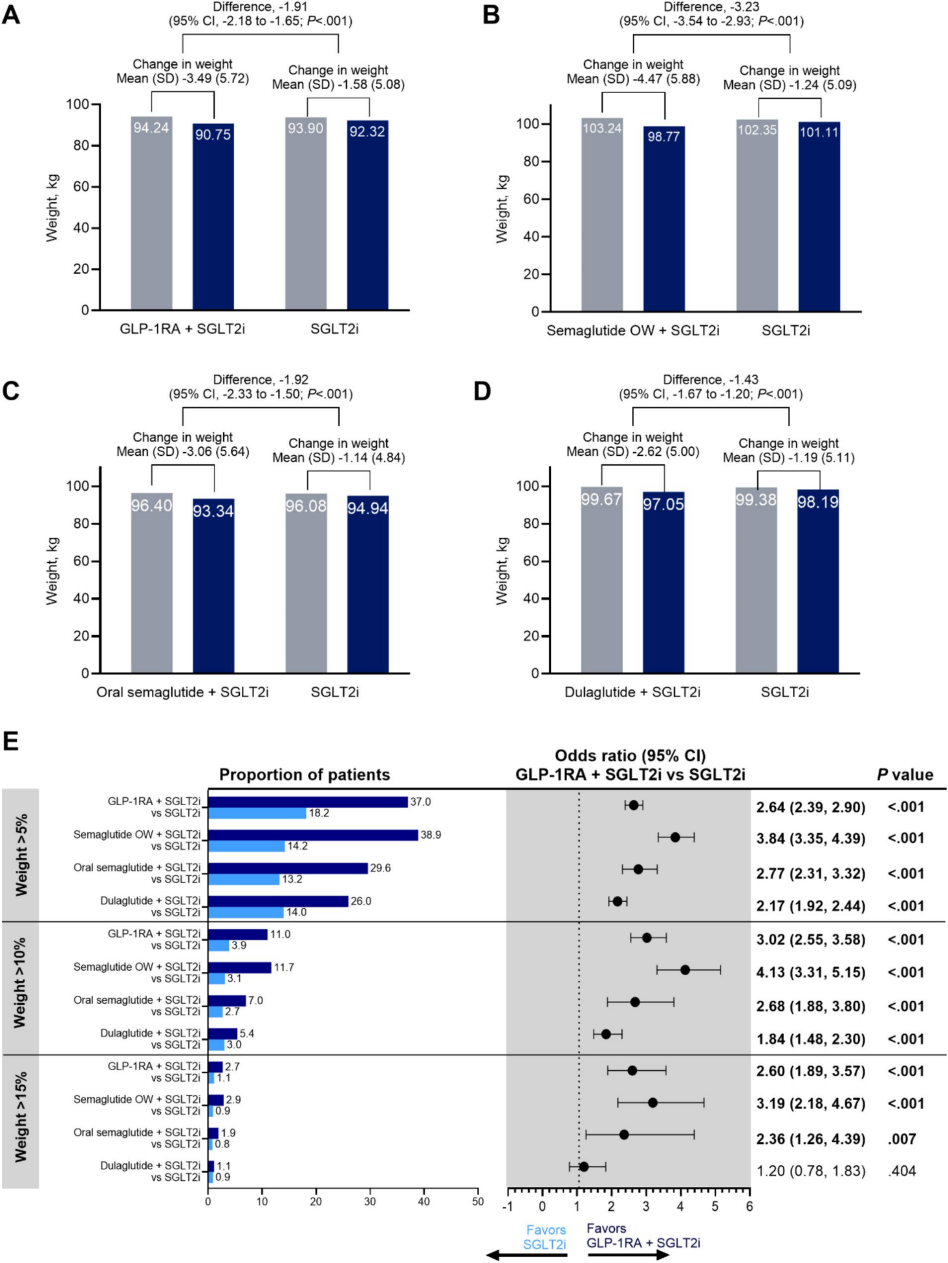
Weighted weight outcomes at 6 months for GLP-1RA and SGLT2i compared with SGLT2i alone. Weighted baseline (gray bars) and 6-month follow-up body weight (dark blue bars) among adults with T2D using **A** combination of GLP-1RA (including semaglutide OW T2D, oral semaglutide, dulaglutide, exenatide OW, and tirzepatide T2D) and SGLT2i compared with SGLT2i alone, **B** combination of semaglutide OW and SGLT2i compared with SGLT2i alone, **C** combination of oral semaglutide and SGLT2i compared with SGLT2i alone, and **D** combination of dulaglutide and SGLT2i compared with SGLT2i alone. **E** Weighted descriptive statistics and odds ratios of achieving weight loss > 5%, > 10%, and > 15% at 6 months among adults with T2D using combination GLP-1RA (including semaglutide OW T2D, oral semaglutide, dulaglutide, exenatide OW, and tirzepatide T2D) and SGLT2i therapy (dark blue bars) compared with SGLT2i alone (light blue bars). Bold odds ratios indicate statistical significance. GLP-1RA indicates glucagonlike peptide-1 receptor agonist; OW, once weekly; SGLT2i, sodium-glucose cotransporter 2 inhibitor; T2D, type 2 diabetes

Compared with those in the SGLT2i group at 6 months, people in the combination group had 1.91 kg greater reduction in weight from baseline (Fig. 3). Combination of SGLT2i with GLP-1RA was significantly associated with 2.33 and 1.87 kg greater reductions in weight from baseline at 12 (See Supplementary Fig. 5, Supplementary Materials) and 18 months (See Supplementary Fig. 6, Supplementary Materials), respectively. Combination therapy was significantly associated with 0.68, 0.83, and 0.67 kg/m^2^ greater reductions in BMI from baseline at 6, 12, and 18 months, respectively (See Supplementary Fig. 7, Supplementary Materials).

People in the combination SGLT2i with semaglutide OW T2D group had 3.23, 3.82, and 3.44 kg greater reductions in weight from baseline at 6 (Fig. 3), 12 (See Supplementary Fig. 5, Supplementary Materials), and 18 months (See Supplementary Fig. 6, Supplementary Materials), respectively. People in the combination SGLT2i with oral semaglutide group had 1.92, 2.13, and 1.92 kg greater reductions in weight from baseline at 6, 12, and 18 months, respectively. People in the combination SGLT2i with dulaglutide group had 1.43, 1.68, and 1.61 kg greater reductions in weight from baseline at 6, 12, and 18 months, respectively.

### Secondary renal outcomes in T2D with CKD cohort

**Fig. 4:**
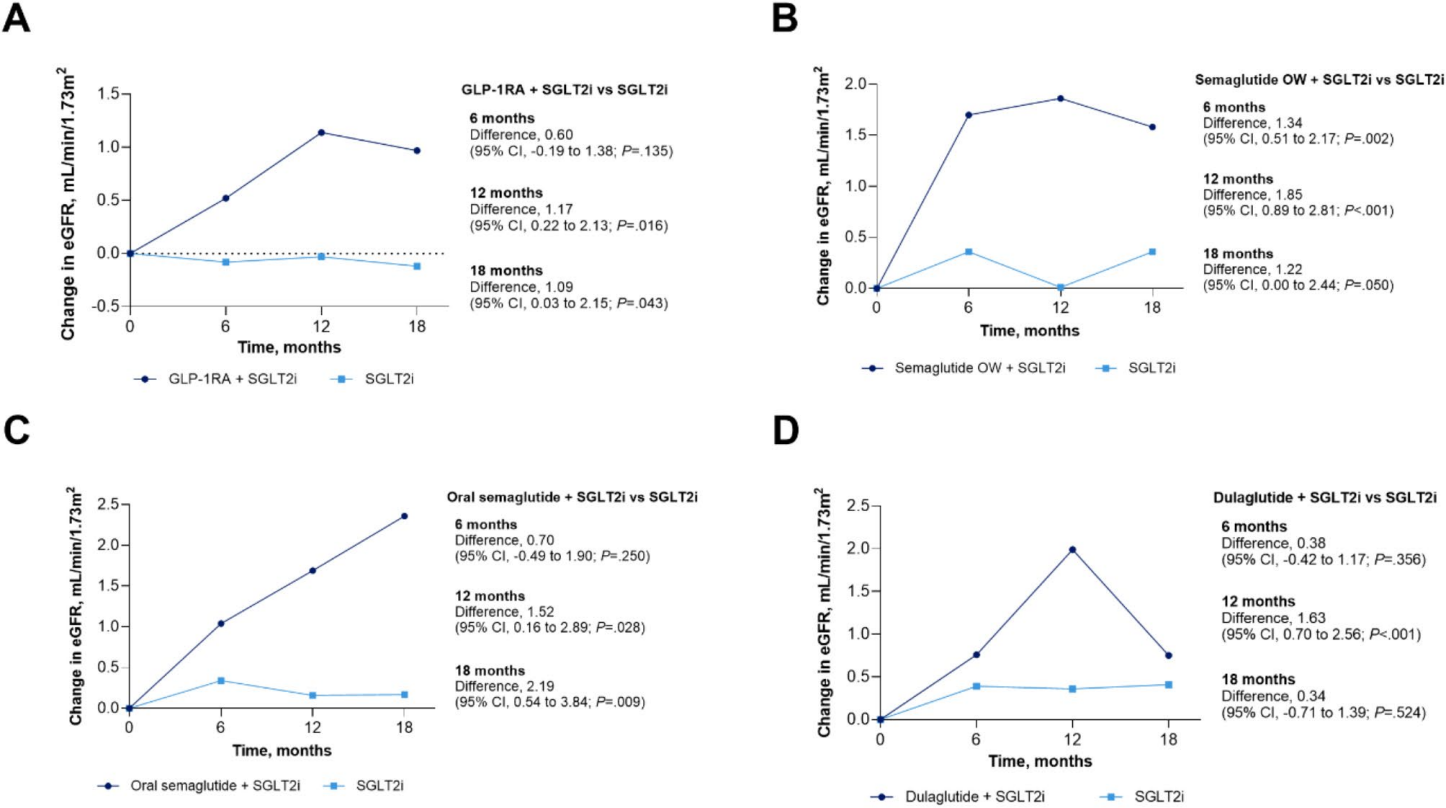
Change in eGFR for GLP-1RA and SGLT2i compared with SGLT2i alone. Change in eGFR from baseline to 6, 12, and 18 months among adults with T2D and CKD using **A** combination of GLP-1RA (including semaglutide OW T2D, oral semaglutide, dulaglutide, exenatide OW, and tirzepatide T2D) and SGLT2i compared with SGLT2i alone, **B** combination of semaglutide OW and SGLT2i compared with SGLT2i alone, **C** combination of oral semaglutide and SGLT2i compared with SGLT2i alone, and **D** combination of dulaglutide and SGLT2i compared with SGLT2i alone. CKD indicates chronic kidney disease; eGFR, estimated glomerular filtration rate; GLP-1RA, glucagonlike peptide-1 receptor agonist; OW, once-weekly; SGLT2i, sodium-glucose cotransporter 2 inhibitor; T2D, type 2 diabetes

Compared with the SGLT2i group, the combination SGLT2i and GLP-1RA group had 1.17 and 1.09 mL/min/1.73 m^2^ greater increases in eGFR from baseline at 12 and 18 months, respectively (Fig. 4, See Supplementary Table 5, Supplementary Materials). Changes in eGFR from baseline were significantly higher at 6, 12, and 18 months (1.34, 1.85, and 1.23 mL/min/1.73 m^2^, respectively) in the combination SGLT2i with semaglutide OW T2D group compared with the SGLT2i group (Fig. 4).

### Sensitivity analyses

Compared with SGLT2i alone, combination therapy in people ≥ 65 years of age was significantly associated with 51%, 33%, 46%, and 45% lower risk of ischemic stroke, MI, 3-point MACE, and 5-point MACE, respectively (See Supplementary Table 6, Supplementary Materials). In people < 65 years of age, greater reductions in risk of ischemic stroke, MI, 3-point, and 5-point MACE with combination therapy were observed. Metabolic and renal outcomes stratified by age are reported in Supplementary Tables 7 and 8, respectively (See Supplementary Materials).

## Discussion

Among US adults with T2D and ASCVD, combination therapy with SGLT2i and GLP-1RA was associated with reduced incidence rates and risks of CV outcomes, including ischemic stroke, MI, 3-point MACE, and 5-point MACE, compared with SGLT2i alone. Combination therapy with SGLT2i and GLP-1RA was also associated with greater reductions in HbA_1c_ and weight in adults with T2D as well as favorable changes in eGFR in adults with T2D and CKD. This real-world study is one of the first to assess CV, metabolic, and renal outcomes associated with newer generation GLP-1RAs (including dual GIP/GLP-1RA) in combination with SGLT2i. Strengths of this study include a large sample size from various payers. This study was carefully designed and used advanced methods, including entropy balancing, to reduce confounding and bias.

Combination of SGLT2i and GLP-1RA was significantly associated with better CV outcomes than SGLT2i alone. Among the individual GLP-1RAs assessed, the largest reductions in risk of CV outcomes were observed with combination therapy with SGLT2i and semaglutide OW T2D. Overall, these findings are consistent with the cardioprotective benefits of combination SGLT2i and GLP-1RA therapy reported in other studies. Post hoc analyses of the EXSCEL and DECLARE-TIMI 58 trials showed reduced risk of MACE, MI, and hospitalization for heart failure with combination GLP-1RA and SGLT2i therapy [31, 32]. Observational analyses in the UK and US found that combination GLP-1RA and SGLT2i therapy was significantly associated with reduced risk of MACE, heart failure, MI, and ischemic stroke [33–36]. Notably, our study showed that combination therapy with SGLT2i and oral semaglutide was associated with significant reductions in risk of MI, 3-point MACE, and 5-point MACE comparable to the other individual GLP-1RAs; however, there was no significant reduction in the risk of ischemic stroke. This may be attributed to inadequate sample size of the combination SGLT2i and oral semaglutide group and warrants further research.

In the present study, significant reductions in HbA_1c_ and weight were observed with combination of SGLT2i and GLP-1RA at 6 months compared with SGLT2i alone; these reductions were sustained to 12 and 18 months. The additive effects of combination SGLT2i and GLP-1RA therapy on HbA_1c_ and weight outcomes have been observed in randomized controlled trials (RCTs), nonrandomized trials, and real-world observational studies. Meta-analyses of RCTs have reported reductions in HbA_1c_ ranging from 0.74 to 0.91% and reductions in body weight ranging from 1.46 kg to 1.95 kg with combination of SGLT2i and GLP-1RA vs. SGLT2i alone [16, 37–39]. Across individual GLP-1RA comparisons, the largest numerical reductions in HbA_1c_ and weight in our study were observed with the combination of SGLT2i and semaglutide OW T2D. Notably, the combination of SGLT2i and oral semaglutide was associated with reductions in HbA_1c_ and weight comparable to the other individual GLP-1RAs. These findings are consistent with post hoc analyses of the PIONEER 4 [40] trial reporting reductions in HbA_1c_ and weight of 1.1% and 5.0 kg, respectively, from baseline to week 52 among patients treated with oral semaglutide (14 mg) with background SGLT2i use.

Evidence of the impact of combination SGLT2i and GLP-1RA therapy on renal outcomes is limited. In the FLOW trial, semaglutide was associated with a reduced risk of kidney failure and a reduction in the decline of eGFR among participants with T2D and CKD; no significant differences were observed between those with or without SGLT2i use at baseline [20]. The proposed mechanisms of kidney benefit with SGLT2i and GLP-1RA therapy are both separate and sometime overlapping [41]. Both drug classes provide metabolic benefits of glycemic control and weight loss and both drug classes may reduce oxidative stress and inflammation in the kidney and elsewhere. SGLT2i have additional hemodynamic and renal energetic effects. Taken together, it is plausible that combination therapy may have additive kidney effects. An actuarial survival analysis estimated the lifetime benefit of SGLT2i, GLP-1RA and nonsteroidal mineralocorticoid receptor agonist (ns-MRA) in people with T2D and albuminuria and found that combination therapy had the lowest hazard ratio of adverse CV and kidney events [42]. An accelerated risk-based implementation of guideline-directed medical therapy for T2D and CKD has also been proposed [43]. In the present study, the absolute change in eGFR was numerically small but occurred over a short period of time and was in favor of the combination. These findings are consistent with the possibility that combination of SGLT2i and GLP-1RA may provide additional renal benefits compared with SGLT2i monotherapy in slowing eGFR decline. A treatment effect of 0.75 mL/min/1.73 m^2^ per year or greater on total slope over chronic slope is considered predictive of a clinical benefit on CKD progress [44]. In the present study, the change in eGFR from baseline to 18 months was 1.09 mL/min/1.73 m^2^ higher with combination therapy GLP-1RA and SGLT2i compared with SGLT2i alone.

Emerging evidence from clinical trials and observational research showing the additive benefits of combining SGLT2i and GLP-1RA, including evidence from the present study, suggests that the addition of GLP-1RA can provide further metabolic and cardiorenal risk reductions in those who are not achieving treatment goals with SGLT2i alone. These findings are also aligned with guideline recommendations to use both SGLT2i and GLP-1RA to treat specific high-risk people. This observational analysis is the first to compare combination therapy of newer generation GLP-1RAs (including dual GLP-1/GIP agonists) and SGLT2is with SGLT2is alone and provides reassurance that the combination is likely more effective for improving cardiorenal and metabolic outcomes in people with T2D. Gradual increases in the use of GLP-1RA and SGLT2i in recent years have been reported; however, the use of combination therapy with GLP-1RA and SGLT2i remains limited despite guideline recommendations [45]. Suboptimal adoption of combination therapy with GLP-1RA and SGLT2i suggests that research on the barriers to guideline adherence is needed and may warrant a call to action in clinical practice change.

## Limitations

This is an observational study and is therefore limited to assessments of associations. Data collection reflects routine clinical practice rather than mandatory assessments at prespecified time points, which may impact the amount of data and their interpretation. Potential measurement errors, missing or unavailable data, and missing clinical details may limit interpretation or applicability of these findings. Some inclusion criteria may have resulted in selection bias; the bias may have selected individuals who better manage their health or receive better care due to the requirements for persistence as well as baseline and follow-up laboratory data. This study prioritized persistence without enforcing strict adherence; additional studies to evaluate the differences in adherence to combination therapy with GLP-1RA and SGLT2i compared with SGLT2i monotherapy and how these differences impact outcomes are needed. Those who used combination therapy may still have had residual selection bias after advanced methods were used to reduce potential bias. Bidirectional comparisons for SGLT2i added to GLP-1RA were not assessed, which may limit comprehensive evaluation of the relative benefits and warrants future research. Limited sample sizes for combination exenatide OW and SGLT2i therapy and combination tirzepatide T2D and SGLT2i therapy precluded individual drug level comparisons vs. SGLT2i alone; future research on combination therapy with these agents is warranted.

## Conclusion

Combination of SGLT2i and newer generation GLP-1RA (including OW GLP-1RAs, once-daily oral semaglutide, and dual GLP-1/GIP agonists) was associated with significantly better results in CV, metabolic, and renal outcomes compared with SGLT2i alone. These findings support the hypothesis that the cardiometabolic benefits of GLP-1RA and SGLT2i may be additive and suggest combination therapy could lead to better clinical outcomes.

## Electronic supplementary material

Below is the link to the electronic supplementary material.

Supplementary material 1.

## Data Availability

The data sets generated and/or analyzed in the current study are not publicly available because they were commercially licensed from the data vendor. Restrictions apply to the availability of these data, which were used under license of this study.

## Abbreviations

ASCVD: Atherosclerotic cardiovascular disease
ATE: Average treatment effect
BMI: Body mass index
CI: Confidence interval
CKD: Chronic kidney disease
CV: Cardiovascular
eGFR: Estimated glomerular filtration rate
GIP: Glucose-dependent insulinotropic polypeptide
GLP-1RA: Glucagonlike peptide-1 receptor agonist
HBA_1c_: Glycated hemoglobin
HR: Hazard ratio
ICD-10-CM: International Classification of Diseases, Tenth Revision, Clinical Modification
MACE: Major adverse cardiovascular event
MI: Myocardial infarction
OW: Once weekly
PY: Person-years
RCTs: Randomized controlled trials
SGLT2i: Sodium-glucose cotransporter 2 inhibitor
SMDs: Standardized mean differences
T2D: Type 2 diabetes
