# Supplementary material for: Real-world effectiveness of adding newer generation GLP-1RA to SGLT2i in type 2 diabetes
Source: Cardiovasc Diabetol. 2025 Apr 24;24:177. doi: 10.1186/s12933-025-02737-1 (PMC12023599; doi:10.1186/s12933-025-02737-1)
Supplement: Supplementary file 1 — Supplementary material 1. [file 12933_2025_2737_MOESM1_ESM.docx]

**Supplementary Material**

Real-World Effectiveness of Adding Newer Generation GLP-1RA to SGLT2i in Type 2 Diabetes

Nathorn Chaiyakunapruk, Xi Tan, Mike Liang, Mico Guevarra, Lin Xie, Alice YY Cheng

**Supplementary Table 1.** Target Variables for Entropy Balancing

**Supplementary Table 2.** Unweighted and Weighted Baseline Characteristics of the T2D With ASCVD Cohort

**Supplementary Table 3.** Unweighted and Weighted Baseline Characteristics of the T2D Cohort

**Supplementary Table 4.** Unweighted and Weighted Baseline Characteristics of the T2D With CKD Cohort

**Supplementary Table 5**. Detailed Weighted Renal Outcomes for GLP-1RA and SGLT2i Compared With SGLT2i Alone

**Supplementary Table 6.** CV Outcomes Stratified by Age Group

**Supplementary Table 7.** HbA_1c_ and Weight Outcomes Stratified by Age Group

**Supplementary Table 8.** Renal Outcomes Stratified by Age Group

**Supplementary Figure 1**. Study Design

**Supplementary Figure 2.** Patient Attrition

**Supplementary Figure 3.** Weighted HbA_1c_ Outcomes at 12 Months for GLP-1RA and SGLT2i Compared With SGLT2i Alone

**Supplementary Figure 4.** Weighted HbA_1c_ Outcomes at 18 Months for GLP-1RA and SGLT2i Compared With SGLT2i Alone

**Supplementary Figure 5.** Weighted Weight Outcomes at 12 Months for GLP-1RA and SGLT2i Compared With SGLT2i Alone

**Supplementary Figure 6.** Weighted Weight Outcomes at 18 Months for GLP-1RA and SGLT2i Compared With SGLT2i Alone

**Supplementary Figure 7.** Weighted BMI Outcomes at 6, 12, and 18 Months for and SGLT2i Compared With SGLT2i Alone

**Supplementary Table 1.** Target Variables for Entropy Balancing

| **Variable** | **Drug class comparisons** | | | **Individual drug comparisons** | | | | | | | | |  |
| --- | --- | --- | --- | --- | --- | --- | --- | --- | --- | --- | --- | --- | --- |
|  | **GLP-1RA + SGLT2i vs SGLT2i** | | | **Semaglutide OW + SGLT2i vs SGLT2i** | | | **Oral semaglutide + SGLT2i vs SGLT2i** | | | **Dulaglutide +  SGLT2i vs SGLT2i** | | |  |
|  | **ASCVD^a^** | **T2D** | **CKD^b^** | **ASCVD^a^** | **T2D** | **CKD^b^** | **ASCVD^a^** | **T2D** | **CKD^b^** | **ASCVD^a^** | **T2D** | **CKD^b^** | |
| Age, continuous spline | X | X | X | X | X | X | X | X | X | X | X | X | |
| Race/ethnicity | X | X | X | X | X | X | X | X | X | X | X | X | |
| Geographical region | X | X | X | X | X | X | X | X | X | X | X | X | |
| Insurance type | X | X | X | X | X | X | X | X | X | X | X | X | |
| Prescriber specialty for index drug | X | X | X | X | X | X | X | X | X | X | X | X | |
| Index year | X | X | ^c^ | X | X | X | X | ^c^ | ^c^ | X | X | X | |
| Months of SGLT2i use in the study period before the index date | X | X | X | X | X | X | X | X | X | X | X | X | |
| Months of SGLT2i use in the whole database before the index date | X | X | X | X | X | X | X | X | X | X | X | X | |
| Months of T2D diagnosis between the first observed T2D diagnosis in the whole dataset available and the index date | X | X | X | X | X | X | X | X | X | X | X | X | |
| Baseline SGLT2i use and adherence | X | X | X | X | X | X | X | X | X | X | X | X | |
| CCI score excluding diabetes, categorical | X | X | X | X | X | X | X | X | X | X | X | X | |
| CCI-MI |  |  |  |  |  |  |  |  | X |  |  |  | |
| CCI-Peptic |  |  |  |  |  |  |  |  | X |  |  |  | |
| CCI-Renal |  |  |  |  |  |  |  |  | X |  |  |  | |
| DCSI score, categorical | X | X | X | X | X | X | X | X | X | X | X | X | |
| DCSI-cardiovascular |  |  |  |  |  |  |  |  | X |  |  |  | |
| DCSI-nephropathy |  |  |  |  |  |  |  |  | X |  |  |  | |
| CKD stage |  |  |  |  |  |  |  |  | X |  |  |  | |
| Number of OP visits at baseline | X | X | X | X | X | X | X | X | X | X | X | X | |
| Number of OP visits 60 days before the index | X | X | X | X | X | X | X | X | X | X | X | X | |
| Number of T2D-related OP visits 60 days before the index |  |  |  | X |  |  | X | X | X |  |  |  | |
| Number of T2D-related IP visits 60 days before the index |  |  |  |  |  |  |  |  | X |  |  |  | |
| **Comorbidities** | | | | | | | | | | | | | |
| Depression | X | X | X | X | X | X | X | X | X | X | X | X | |
| Acute/chronic pancreatitis | X | X | X | X | X | X | X | X | X | X | X | X | |
| Obesity | X | X | X | X | X | X | X | X | X | X | X | X | |
| GERD |  |  |  |  |  |  |  |  | X |  |  |  | |
| **Medications** | | | | | | | | | | | | | |
| Number of GLTs used | X | X | X | X | X | X | X | X | X | X | X | X | |
| Metformin | X | X | X | X | X | X | X | X | X | X | X | X | |
| Sulfonylurea | X | X | X | X | X | X | X | X | X | X | X | X | |
| DPP-4i | X | X | X | X | X | X | X | X | X | X | X | X | |
| Basal insulin | X | X | X | X | X | X | X | X | X | X | X | X | |
| Other insulin | X | X | X | X | X | X | X | X | X | X | X | X | |
| Other diuretics |  |  |  |  |  |  |  |  | X |  |  |  | |
| **Procedures** | | | | | | | | | | | | | |
| PCI |  |  |  |  |  |  |  |  | X |  |  |  | |
| **HbA_1c_ and BMI measures** | | | | | | | | | | | | | |
| Baseline HbA_1c_, continuous spline |  | X |  |  | X |  |  | X |  |  | X |  | |
| Baseline BMI, continuous spline |  | X |  |  | X |  |  | X |  |  | X |  | |
| Baseline HbA_1c_, categorical | X |  | X | X |  | X | X | ^d^ | X | X |  | X | |
| Baseline BMI, categorical | X |  | X | X |  | X | X | ^d^ | X | X |  | X | |
| **T2D with ASCVD cohort** | | | | | | | | | | | | | |
| Months of ASCVD diagnosis between the first observed ASCVD diagnosis in the whole dataset available and the index date, continuous spline | X |  |  | X |  |  | X |  |  | X |  |  | |
| **T2D with CKD cohort** | | | | | | | | | | | | | |
| Months of CKD diagnosis between the first observed CKD diagnosis in the whole dataset available and the index date, continuous spline |  |  | X |  |  | X |  |  | X |  |  | X | |

ASCVD indicates atherosclerotic cardiovascular disease; BMI, body mass index; CCI, Charlson Comorbidity Index; CKD, chronic kidney disease; DCSI, Diabetes Complications Severity Index; DPP-4i, dipeptidyl peptidase-4 inhibitors; GERD, gastroesophageal reflux disease; GLP-1RA, glucagonlike peptide-1 receptor agonist; GLT, glucose-lowering therapy; HbA_1c_, glycated hemoglobin; IP, inpatient; MI, myocardial infarction; OP, outpatient; PCI, percutaneous coronary intervention; SGLT2i, sodium-glucose cotransporter 2 inhibitor; T2D, type 2 diabetes.

^a^ T2D with ASCVD cohort.

^b^ T2D with CKD cohort.

^c^Index time as continuous spline was used instead of categorical index year.

^d^HbA_1c_ and BMI category distributions were not balanced with only continuous HbA_1c_ and B

**Supplementary Table 2.** Unweighted and Weighted Baseline Characteristics of the T2D With ASCVD Cohort

|  |  | | **Unweighted** | | | | **Weighted** | | | |
| --- | --- | --- | --- | --- | --- | --- | --- | --- | --- | --- |
| **Characteristic** |  | **SGLT2i + GLP-1RA** | | **SGLT2i** | **SMD** | **SGLT2i +  GLP-1RA** | | **SGLT2i** | **SMD** |  |
| N |  | 34 690 | | 130 220 |  | 34 690 | | 130 220 |  |  |
| Age, y, mean (SD) |  | 60.24 (9.35) | | 64.62 (10.54) | **0.439** | 63.70 (10.45) | | 63.70 (10.46) | <0.001 |  |
| Age group, y, n (%) | 18-44 | 1592 (4.6) | | 3759 (2.9) | **0.423** | 1093 (3.2) | | 4268 (3.3) | 0.008 |  |
|  | 45-64 | 23 547 (67.9) | | 66 757 (51.3) |  | 19 008 (54.8) | | 71 307 (54.8) |  |  |
|  | 65-79 | 8634 (24.9) | | 47 827 (36.7) |  | 11 914 (34.3) | | 44 531 (34.2) |  |  |
|  | 80+ | 917 (2.6) | | 11 877 (9.1) |  | 2675 (7.7) | | 10 114 (7.8) |  |  |
| Sex, n (%) | Male | 21 137 (60.9) | | 81 196 (62.4) | 0.046 | 20 604 (59.4) | | 81 577 (62.6) | 0.072 |  |
|  | Female | 13 147 (37.9) | | 47 036 (36.1) |  | 13 646 (39.3) | | 46 724 (35.9) |  |  |
|  | Unknown | 406 (1.2) | | 1988 (1.5) |  | 440 (1.3) | | 1919 (1.5) |  |  |
| Insurance type, n (%) | Commercial | 17 931 (51.7) | | 47 165 (36.2) | **0.357** | 13 693 (39.5) | | 51 403 (39.5) | 0.001 |  |
|  | Medicare | 9753 (28.1) | | 57 142 (43.9) |  | 14 072 (40.6) | | 52 819 (40.6) |  |  |
|  | Medicaid | 6963 (20.1) | | 25 739 (19.8) |  | 6879 (19.8) | | 25 824 (19.8) |  |  |
|  | Unknown | 43 (0.1) | | 174 (0.1) |  | 46 (0.1) | | 174.5 (0.1) |  |  |
| Geographical region, n (%) | Northeast | 8920 (25.7) | | 36 068 (27.7) | **0.148** | 9464 (27.3) | | 35 523 (27.3) | 0.001 |  |
|  | South | 13 132 (37.9) | | 43 666 (33.5) |  | 11 946 (34.4) | | 44 853 (34.4) |  |  |
|  | Midwest | 6805 (19.6) | | 25 416 (19.5) |  | 6778 (19.5) | | 25 442 (19.5) |  |  |
|  | West | 5736 (16.5) | | 23 328 (17.9) |  | 6113 (17.6) | | 22 950 (17.6) |  |  |
|  | Unknown | 97 (0.3) | | 1742 (1.3) |  | 389 (1.1) | | 1452 (1.1) |  |  |
| Race/ethnicity, n (%) | White | 16 010 (46.2) | | 58 095 (44.6) | **0.172** | 15 590 (44.9) | | 58 517 (44.9) | <0.001 |  |
|  | Black | 3711 (10.7) | | 17 044 (13.1) |  | 4367 (12.6) | | 16 389 (12.6) |  |  |
|  | Hispanic | 5894 (17.0) | | 23 911 (18.4) |  | 6270 (18.1) | | 23 534 (18.1) |  |  |
|  | Asian | 1944 (5.6) | | 10 658 (8.2) |  | 2651 (7.6) | | 9947 (7.6) |  |  |
|  | Other | 1483 (4.3) | | 4757 (3.7) |  | 1312 (3.8) | | 4929 (3.8) |  |  |
|  | Unknown | 5648 (16.3) | | 15 755 (12.1) |  | 4501 (13.0) | | 16 904.4 (13.0) |  |  |
| Index year, n (%) | 2018 | 1937 (5.6) | | 9371 (7.2) | **0.198** | 2379 (6.9) | | 8929 (6.9) | 0.007 |  |
|  | 2019 | 3816 (11.0) | | 9934 (7.6) |  | 2893 (8.3) | | 10 854 (8.3) |  |  |
|  | 2020 | 5439 (15.7) | | 16 359 (12.6) |  | 4586 (13.2) | | 17 206 (13.2) |  |  |
|  | 2021 | 9389 (27.1) | | 32 097 (24.6) |  | 8727 (25.2) | | 32 761 (25.2) |  |  |
|  | 2022 | 14 108 (40.7) | | 62 348 (47.9) |  | 16 084 (46.4) | | 60 368 (46.4) |  |  |
|  | 2023 | 1 (<0.1) | | 111 (0.1) |  | 21 (0.1) | | 102 (0.1) |  |  |
| Months of SGLT2i use in the study period before the index date, mean (SD) |  | 17.35 (15.61) | | 9.87 (14.11) | **0.503** | 11.45 (14.81) | | 11.45 (14.75) | <0.001 |  |
| Months of SGLT2i use in the whole database before the index date, mean (SD) |  | 23.13 (21.31) | | 13.22 (18.83) | **0.493** | 15.31 (19.83) | | 15.31 (19.79) | <0.001 |  |
| Months of T2D diagnosis between the first observed T2D diagnosis in the whole dataset available and the index date, mean (SD) |  | 54.78 (19.47) | | 53.04 (22.08) | 0.084 | 53.41 (21.57) | | 53.41 (21.57) | <0.001 |  |
| Months of ASCVD diagnosis between the first observed ASCVD diagnosis in the whole dataset available and the index date, mean (SD) |  | 35.82 (21.79) | | 36.65 (22.74) | 0.037 | 36.48 (22.54) | | 36.48 (22.55) | <0.001 |  |
| Baseline HbA_1c_, %, mean (SD) |  | 8.51 (1.60) | | 7.79 (1.64) | **0.444** | 7.98 (1.62) | | 7.96 (1.68) | 0.014 |  |
| Baseline HbA_1c_, categorical, n (%) | <7% | 1170 (3.4) | | 9650 (7.4) | **0.283** | 2276 (6.6) | | 8546 (6.6) | <0.001 |  |
|  | 7% to <8% | 2456 (7.1) | | 8465 (6.5) |  | 2297 (6.6) | | 8630 (6.6) |  |  |
|  | 8% to <9% | 2478 (7.1) | | 5082 (3.9) |  | 1590 (4.6) | | 5970 (4.6) |  |  |
|  | 9% to <10% | 1367 (3.9) | | 2660 (2.0) |  | 847 (2.4) | | 3181 (2.4) |  |  |
|  | ≥10% | 1554 (4.5) | | 2928 (2.2) |  | 944 (2.7) | | 3535 (2.7) |  |  |
|  | Unknown | 25 665 (74.0) | | 101 435 (77.9) |  | 26 737 (77.1) | | 100 359 (77.1) |  |  |
| Baseline BMI, kg/m^2^, mean (SD) |  | 34.12 (6.06) | | 31.64 (6.15) | **0.407** | 32.27 (6.27) | | 32.20 (6.19) | 0.012 |  |
| Baseline BMI, categorical, n (%) | Normal or underweight | 819 (2.4) | | 6238 (4.8) | **0.288** | 1485 (4.3) | | 5571 (4.3) | <0.001 |  |
|  | Overweight | 2906 (8.4) | | 14 474 (11.1) |  | 3656 (10.5) | | 13 723 (10.5) |  |  |
|  | Obesity class 1 | 4752 (13.7) | | 16 220 (12.5) |  | 4411 (12.7) | | 16 559 (12.7) |  |  |
|  | Obesity class 2 | 4067 (11.7) | | 9954 (7.6) |  | 2950 (8.5) | | 11 072 (8.5) |  |  |
|  | Obesity class 3 | 3969 (11.4) | | 7874 (6.0) |  | 2492 (7.2) | | 9356 (7.2) |  |  |
|  | Unknown | 18 177 (52.4) | | 75 460 (57.9) |  | 19 696 (56.8) | | 73 940 (56.8) |  |  |
| Patients with weight measurements, n (%) |  | 6664 (19.2) | | 22 751 (17.5) | 0.045 | 6050 (17.4) | | 23 292 (17.9) | 0.012 |  |
| Baseline weight, kg, mean (SD) |  | 100.67 (23.29) | | 91.69 (22.38) | **0.393** | 94.38 (23.06) | | 93.48 (22.76) | 0.039 |  |
| Prescriber specialty for index drug, n (%) | Primary | 23 814 (68.6) | | 95 447 (73.3) | **0.448** | 25 087 (72.3) | | 94 175 (72.3) | <0.001 |  |
|  | Endocrinology | 7820 (22.5) | | 11 545 (8.9) |  | 4073 (11.7) | | 15 287 (11.7) |  |  |
|  | Other | 1907 (5.5) | | 17 358 (13.3) |  | 4053 (11.7) | | 15 208 (11.7) |  |  |
|  | Unknown | 1149 (3.3) | | 5870 (4.5) |  | 1477 (4.3) | | 5550 (4.3) |  |  |
| Index drug, n (%) | Semaglutide OW T2D | 12 145 (35.0) | | - | **-** | 11 279.2 (32.5) | | - | **-** |  |
|  | Oral semaglutide | 4041 (11.6) | | - |  | 4317.0 (12.4) | | - |  |  |
|  | Tirzepatide T2D | 789 (2.3) | | - |  | 691.7 (2.0) | | - |  |  |
|  | Dulaglutide | 16 202 (46.7) | | - |  | 16 807.5 (48.5) | | - |  |  |
|  | Exenatide ER | 1456 (4.2) | | - |  | 1529.7 (4.4) | | - |  |  |
|  | Multiple | 57 (0.2) | | - |  | 64.9 (0.2) | | - |  |  |
| Baseline SGLT2i use and adherence, n (%) | <1 y SGLT2i use | 14 385 (41.5) | | 87 695 (67.3) | **0.550** | 21 475 (61.9) | | 80 607 (61.9) | <0.001 |  |
|  | SGLT2i PDC <50% | 1105 (3.2) | | 3594 (2.8) |  | 989 (2.8) | | 3707 (2.8) |  |  |
|  | SGLT2i PDC <80% | 759 (2.2) | | 2624 (2.0) |  | 711 (2.1) | | 2669 (2.0) |  |  |
|  | SGLT2i PDC ≥80% | 18 441 (53.2) | | 36 307 (27.9) |  | 11 515 (33.2) | | 43 238 (33.2) |  |  |
| Number of GLTs used, n (%) | 0 | 2079 (6.0) | | 15 908 (12.2) | **0.336** | 3782 (10.9) | | 14 197 (10.9) | <0.001 |  |
|  | 1 | 10 009 (28.9) | | 47 356 (36.4) |  | 12 068 (34.8) | | 45 295 (34.8) |  |  |
|  | 2 | 12 039 (34.7) | | 41 456 (31.8) |  | 11 252 (32.4) | | 42 243 (32.4) |  |  |
|  | 3+ | 10 563 (30.4) | | 25 500 (19.6) |  | 7588 (21.9) | | 28 485 (21.9) |  |  |
| Baseline GLT use, n (%) | Metformin | 26 108 (75.3) | | 91 463 (70.2) | **0.113** | 24 733 (71.3) | | 92 840 (71.3) | <0.001 |  |
|  | Sulfonylurea | 10 757 (31.0) | | 35 504 (27.3) | 0.082 | 9730 (28.0) | | 36 533 (28.1) | <0.001 |  |
|  | DPP-4i | 11 460 (33.0) | | 32 656 (25.1) | **0.176** | 9278 (26.7) | | 34 842 (26.8) | <0.001 |  |
|  | TZD | 2833 (8.2) | | 7779 (6.0) | 0.086 | 2393 (6.9) | | 8203 (6.3) | 0.024 |  |
|  | Basal insulin | 10 851 (31.3) | | 27 834 (21.4) | **0.226** | 8138 (23.5) | | 30 547 (23.5) | <0.001 |  |
|  | Other insulin | 6175 (17.8) | | 15 592 (12.0) | **0.164** | 4579 (13.2) | | 17 186 (13.2) | <0.001 |  |
|  | AGI | 220 (0.6) | | 638 (0.5) | 0.019 | 197 (0.6) | | 6567 (0.5) | 0.009 |  |
|  | MEG | 552 (1.6) | | 1573 (1.2) | 0.033 | 497 (1.4) | | 1640 (1.3) | 0.015 |  |
| CCI score excluding diabetes, mean (SD) |  | 1.57 (1.64) | | 1.92 (1.87) | **0.195** | 1.84 (1.83) | | 1.84 (1.83) | <0.001 |  |
| CCI score excluding diabetes, categorical, n (%) | 0-1 | 20 274 (58.4) | | 65 720 (50.5) | **0.193** | 18 087 (52.1) | | 67 907 (52.1) | 0.001 |  |
|  | 2 | 6571 (18.9) | | 25 469 (19.6) |  | 6740 (19.4) | | 25 297 (19.4) |  |  |
|  | 3 | 3749 (10.8) | | 16 867 (13.0) |  | 4336 (12.5) | | 16 280 (12.5) |  |  |
|  | 4 | 2101 (6.1) | | 10 189 (7.8) |  | 2585 (7.5) | | 9704 (7.5) |  |  |
|  | 5 | 1008 (2.9) | | 5466 (4.2) |  | 1361 (3.9) | | 5113 (3.9) |  |  |
|  | 6+ | 987 (2.8) | | 6509 (5.0) |  | 1581 (4.6) | | 5920 (4.5) |  |  |
| DCSI score, mean (SD) |  | 2.48 (1.89) | | 2.65 (1.94) | 0.089 | 2.62 (1.93) | | 2.62 (1.93) | <0.001 |  |
| DCSI score, categorical, n (%) | 0 | 5107 (14.7) | | 17 259 (13.3) | 0.095 | 4705 (13.6) | | 17 661 (13.6) | <0.001 |  |
|  | 1 | 6722 (19.4) | | 22 378 (17.2) |  | 6122 (17.6) | | 22 981 (17.6) |  |  |
|  | 2 | 7833 (22.6) | | 29 131 (22.4) |  | 7776 (22.4) | | 29 189 (22.4) |  |  |
|  | 3 | 5985 (17.3) | | 22 819 (17.5) |  | 6060 (17.5) | | 22 743 (17.5) |  |  |
|  | 4 | 3957 (11.4) | | 16 533 (12.7) |  | 4309 (12.4) | | 16 179 (12.4) |  |  |
|  | 5 | 2454 (7.1) | | 10 661 (8.2) |  | 2759 (8.0) | | 10 358 (8.0) |  |  |
|  | 6+ | 2632 (7.6) | | 11 439 (8.8) |  | 2960 (8.5) | | 11 110 (8.5) |  |  |
| Comorbidities, n (%) | Hypertension | 31 364 (90.4) | | 117 340 (90.1) | 0.010 | 31 391 (90.5) | | 117 088 (89.9) | 0.019 |  |
|  | Hyperlipidemia | 30 699 (88.5) | | 113 067 (86.8) | 0.051 | 30 109 (86.8) | | 113 308 (87.0) | 0.006 |  |
|  | Atrial fibrillation and flutter | 3780 (10.9) | | 19 554 (15.0) | **0.123** | 4414 (12.7) | | 18 546 (14.2) | 0.044 |  |
|  | HF | 5766 (16.6) | | 29 936 (23.0) | **0.160** | 6878 (19.8) | | 28 256 (21.7) | 0.046 |  |
|  | Depression | 7437 (21.4) | | 25 294 (19.4) | 0.050 | 6886 (19.8) | | 25 847 (19.8) | <0.001 |  |
|  | Anxiety | 6658 (19.2) | | 21 816 (16.8) | 0.064 | 6184 (17.8) | | 22 373 (17.2) | 0.017 |  |
|  | Smoking | 5432 (15.7) | | 21 065 (16.2) | 0.014 | 5447 (15.7) | | 21 226 (16.3) | 0.016 |  |
|  | Acute/chronic pancreatitis | 312 (0.9) | | 2221 (1.7) | 0.071 | 532 (1.5) | | 2000 (1.5) | <0.001 |  |
|  | MI | 6019 (17.4) | | 26 132 (20.1) | 0.070 | 6085 (17.5) | | 25 810 (19.8) | 0.058 |  |
|  | Ischemic stroke | 4717 (13.6) | | 21 373 (16.4) | 0.079 | 5336 (15.4) | | 20 967 (16.1) | 0.020 |  |
|  | PAD | 15 451 (44.5) | | 64 042 (49.2) | 0.093 | 16 778 (48.4) | | 62 932 (48.3) | 0.001 |  |
|  | TIA | 3671 (10.6) | | 15 294 (11.7) | 0.037 | 3941 (11.4) | | 15 093 (11.6) | 0.007 |  |
|  | ACS | 9955 (28.7) | | 43 252 (33.2) | 0.098 | 11 128 (32.1) | | 42 235 (32.4) | 0.008 |  |
|  | Other CHD | 25 134 (72.5) | | 95 309 (73.2) | 0.017 | 25 431 (73.3) | | 94 875 (72.9) | 0.010 |  |
|  | Obesity | 19 601 (56.5) | | 56 465 (43.4) | **0.265** | 16 001 (46.1) | | 60 067 (46.1) | <0.001 |  |
|  | Anemia | 6705 (19.3) | | 31 330 (24.1) | **0.115** | 7869 (22.7) | | 30 159 (23.2) | 0.011 |  |
|  | Arrhythmia | 10 779 (31.1) | | 46 767 (35.9) | **0.103** | 11 530 (33.2) | | 45 546 (35.0) | 0.037 |  |
|  | Asthma | 9949 (28.7) | | 32 766 (25.2) | 0.079 | 9503 (27.4) | | 33 338 (25.6) | 0.041 |  |
|  | GERD | 10 037 (28.9) | | 36 233 (27.8) | 0.025 | 10 100 (29.1) | | 36 091 (27.7) | 0.031 |  |
|  | Musculoskeletal pain | 18 017 (51.9) | | 64 732 (49.7) | 0.045 | 17 892 (51.6) | | 64 933 (49.9) | 0.034 |  |
|  | MASH | 3762 (10.8) | | 11 384 (8.7) | 0.071 | 3608 (10.4) | | 11 686 (9.0) | 0.048 |  |
| Diseases/conditions related to weight changes, n (%) | Cancer | 4776 (13.8) | | 21 773 (16.7) | 0.082 | 5399 (15.6) | | 21 115 (16.2) | 0.018 |  |
|  | Cirrhosis | 586 (1.7) | | 2737 (2.1) | 0.030 | 659 (1.9) | | 2727 (2.1) | 0.014 |  |
|  | CKD | 155 (0.4) | | 836 (0.6) | 0.027 | 248 (0.7) | | 776 (0.6) | 0.015 |  |
|  | Dialysis | 255 (0.7) | | 1274 (1.0) | 0.026 | 361 (1.0) | | 1201 (0.9) | 0.012 |  |
|  | Eating disorders | 279 (0.8) | | 1366 (1.0) | 0.026 | 331 (1.0) | | 1305 (1.0) | 0.005 |  |
|  | Feeding disorders | 47 (0.1) | | 202 (0.2) | 0.005 | 43 (0.1) | | 191 (0.1) | 0.006 |  |
|  | HIV | 257 (0.7) | | 825 (0.6) | 0.013 | 320 (0.9) | | 794 (0.6) | 0.036 |  |
| Procedures, n (%) | PCI | 500 (1.4) | | 2251 (1.7) | 0.023 | 477 (1.4) | | 2199 (1.7) | 0.026 |  |
|  | CABG | 315 (0.9) | | 1386 (1.1) | 0.016 | 298 (0.8) | | 1382 (1.1) | 0.023 |  |
|  | Major amputation | 101 (0.3) | | 400 (0.3) | 0.003 | 75 (0.2) | | 424 (0.3) | 0.021 |  |
|  | Bariatric | 83 (0.2) | | 401 (0.3) | 0.013 | 85 (0.2) | | 406 (0.3) | 0.013 |  |
|  | Dialysis | 1872 (5.4) | | 8335 (6.4) | 0.043 | 2052 (5.9) | | 7959 (6.1) | 0.008 |  |
|  | Percutaneous intervention-carotid | 120 (0.3) | | 573 (0.4) | 0.015 | 149 (0.4) | | 557 (0.4) | <0.001 |  |
|  | Percutaneous intervention-peripheral arteries | 365 (1.1) | | 1445 (1.1) | 0.006 | 381 (1.1) | | 1420 (1.1) | 0.001 |  |
|  | Thrombectomy/ thrombolysis | 251 (0.7) | | 1183 (0.9) | 0.021 | 252 (0.7) | | 1170 (0.9) | 0.019 |  |
| Number of OP visits at baseline, mean (SD) |  | 32.70 (46.03) | | 33.15 (48.30) | 0.010 | 33.06 (46.17) | | 33.06 (48.39) | <0.001 |  |
| Number of IP visits at baseline, mean (SD) |  | 0.24 (0.70) | | 0.35 (0.99) | **0.119** | 0.28 (0.80) | | 0.34 (0.96) | 0.067 |  |
| Number of T2D-related OP visits at baseline, mean (SD) |  | 9.27 (17.31) | | 8.33 (17.43) | 0.054 | 8.82 (16.20) | | 8.45 (17.55) | 0.022 |  |
| Number of T2D-related IP visits at baseline, mean (SD) |  | 0.17 (0.54) | | 0.21 (0.64) | 0.062 | 0.19 (0.60) | | 0.21 (0.64) | 0.039 |  |
| Number of OP visits 60 days before the index, mean (SD) |  | 6.09 (8.44) | | 6.10 (9.01) | 0.001 | 6.10 (8.29) | | 6.10 (9.10) | <0.001 |  |
| Number of IP visits 60 days before the index, mean (SD) |  | 0.04 (0.21) | | 0.06 (0.28) | 0.091 | 0.04 (0.23) | | 0.06 (0.28) | 0.049 |  |
| Number of T2D-related OP visits 60 days before the index, mean (SD) |  | 2.01 (3.47) | | 1.68 (3.50) | 0.097 | 1.95 (3.28) | | 1.68 (3.55) | 0.077 |  |
| Number of T2D-related IP visits 60 days before the index, mean (SD) |  | 0.03 (0.17) | | 0.04 (0.21) | 0.053 | 0.03 (0.19) | | 0.04 (0.21) | 0.024 |  |
| Medications, n (%) | Anticoagulants | 4286 (12.4) | | 20 818 (16.0) | **0.104** | 4727 (13.6) | | 20 061 (15.4) | 0.051 |  |
|  | Antidepressants | 12 122 (34.9) | | 38 208 (29.3) | **0.120** | 11 424 (32.9) | | 38 880 (29.9) | 0.066 |  |
|  | Antihyperlipidemic agent | 31 556 (91.0) | | 115 945 (89.0) | 0.064 | 30 709 (88.5) | | 116 068 (89.1) | 0.019 |  |
|  | Antihypertensive agent | 32 073 (92.5) | | 120 485 (92.5) | 0.003 | 31 732 (91.5) | | 120 237 (92.3) | 0.032 |  |
|  | Antiplatelets | 6500 (18.7) | | 26 286 (20.2) | 0.037 | 6502 (18.7) | | 26 126 (20.1) | 0.033 |  |
|  | ACE | 15 750 (45.4) | | 55 982 (43.0) | 0.049 | 14 733 (42.5) | | 56 675 (43.5) | 0.021 |  |
|  | ARB | 13 537 (39.0) | | 52 611 (40.4) | 0.028 | 13 831 (39.9) | | 51 636 (39.7) | 0.004 |  |
|  | Beta blocker | 19 280 (55.6) | | 76 060 (58.4) | 0.057 | 19 486 (56.2) | | 75 159 (57.7) | 0.031 |  |
|  | Loop diuretics | 6648 (19.2) | | 29 304 (22.5) | 0.082 | 7319 (21.1) | | 28 178 (21.6) | 0.013 |  |
|  | MRA | 2946 (8.5) | | 13 770 (10.6) | 0.071 | 3156 (9.1) | | 13 151 (10.1) | 0.034 |  |
|  | Nitrates | 2855 (8.2) | | 12 581 (9.7) | 0.050 | 3148 (9.1) | | 12 186 (9.4) | 0.010 |  |
|  | Other HF medications | 1624 (4.7) | | 10 315 (7.9) | **0.134** | 1930 (5.6) | | 9557 (7.3) | 0.072 |  |
|  | Other diuretics | 10 429 (30.1) | | 35 096 (27.0) | 0.069 | 10 383 (29.9) | | 34 958 (26.8) | 0.068 |  |
|  | Medications with weight gain/loss | 28 062 (80.9) | | 100 081 (76.9) | 0.099 | 27 029 (77.9) | | 100 828 (77.4) | 0.012 |  |

ACE indicates angiotensin-converting enzyme; ACS, acute coronary syndrome; AGI, alpha-glucosidase inhibitor; ARB, angiotensin receptor blocker; ASCVD, atherosclerotic cardiovascular disease; BMI, body mass index; CABG, coronary artery bypass grafting; CCI, Charlson Comorbidity Index; CHD, coronary heart disease; CKD, chronic kidney disease; DCSI, Diabetes Complications Severity Index; DPP-4i, dipeptidyl peptidase-4 inhibitor; ER, extended release; GERD, gastroesophageal reflux disease; GLP-1RA, glucagonlike peptide-1 receptor agonist; GLT, glucose-lowering therapy; HbA_1c_, glycated hemoglobin; HF, heart failure; IP, inpatient; MASH, metabolic dysfunction-associated steatohepatitis; MEG, meglitinide; MI, myocardial infarction; MRA, mineralocorticoid receptor agonist; OP, outpatient; OW, once weekly; PAD, peripheral artery disease; PCI, percutaneous coronary intervention; PDC, proportion of days covered; SGLT2i, sodium-glucose cotransporter 2 inhibitor; SMD, standardized mean difference; T2D, type 2 diabetes; TIA, transient ischemic attack; TZD, thiazolidinedione.

Bold font indicates statistical significance (SMD ≥0.1).

**Supplementary Table 3.** Unweighted and Weighted Baseline Characteristics of the T2D Cohort

| **Characteristic** | | **Unweighted** | | | **Weighted** | | |
| --- | --- | --- | --- | --- | --- | --- | --- |
|  |  | **SGLT2i +  GLP-1RA** | **SGLT2i** | **SMD** | **SGLT2i + GLP-1RA** | **SGLT2i** | **SMD** |
| N |  | 8220 | 22 891 |  | 8220 | 22 891 |  |
| Age, y, mean (SD) |  | 58.11 (10.05) | 62.26 (11.36) | **0.387** | 61.17 (11.16) | 61.16 (11.18) | <0.001 |
| Age group, y, n (%) | 18-44 | 746 (9.1) | 1446 (6.3) | **0.414** | 591 (7.2) | 1635 (7.1) | 0.016 |
|  | 45-64 | 5638 (68.6) | 12 254 (53.5) |  | 4731 (57.6) | 13 096 (57.2) |  |
|  | 65-79 | 1683 (20.5) | 7661 (33.5) |  | 2480 (30.2) | 6916 (30.2) |  |
|  | 80+ | 153 (1.9) | 1530 (6.7) |  | 418 (5.1) | 1244 (5.4) |  |
| Sex, n (%) | Male | 4960 (60.3) | 14 043 (61.3) | 0.022 | 4780 (58.2) | 14 150 (61.8) | 0.076 |
|  | Female | 3172 (38.6) | 8590 (37.5) |  | 3349 (40.7) | 8483 (37.1) |  |
|  | Unknown | 88 (1.1) | 258 (1.1) |  | 91 (1.1) | 258 (1.1) |  |
| Insurance type, n (%) | Commercial | 5196 (63.2) | 10 325 (45.1) | **0.390** | 4100 (49.9) | 11 421 (49.9) | 0.001 |
|  | Medicare | 1908 (23.2) | 8930 (39.0) |  | 2864 (34.8) | 7975 (34.8) |  |
|  | Medicaid | 1105 (13.4) | 3546 (15.5) |  | 1229 (14.9) | 3423 (15.0) |  |
|  | Unknown | 11 (0.1) | 90 (0.4) |  | 27 (0.3) | 73 (0.3) |  |
| Geographic region, n (%) | Northeast | 2478 (30.1) | 6521 (28.5) | **0.159** | 2376 (28.9) | 6621 (28.9) | <0.001 |
|  | South | 3124 (38.0) | 7616 (33.3) |  | 2839 (34.5) | 7903 (34.5) |  |
|  | Midwest | 1102 (13.4) | 3147 (13.7) |  | 1123 (13.7) | 3126 (13.7) |  |
|  | West | 1512 (18.4) | 5567 (24.3) |  | 1871 (22.8) | 5208 (22.8) |  |
|  | Unknown | 4 (<0.1) | 40 (0.2) |  | 12 (0.1) | 34 (0.1) |  |
| Race/ethnicity, n (%) | White | 4914 (59.8) | 11 962 (52.3) | **0.206** | 4457 (54.2) | 12 417 (54.2) | 0.001 |
|  | Black | 818 (10.0) | 2541 (11.1) |  | 888 (10.8) | 2472 (10.8) |  |
|  | Hispanic | 1329 (16.2) | 4343 (19.0) |  | 1500 (18.2) | 4173 (18.2) |  |
|  | Asian | 559 (6.8) | 2632 (11.5) |  | 843 (10.3) | 2348 (10.3) |  |
|  | Other | 236 (2.9) | 567 (2.5) |  | 212 (2.6) | 590 (2.6) |  |
|  | Unknown | 364 (4.4) | 846 (3.7) |  | 321 (3.9) | 892 (3.9) |  |
| Index year, n (%) | 2018 | 756 (9.2) | 2606 (11.4) | **0.243** | 889 (10.8) | 2473 (10.8) | 0.029 |
|  | 2019 | 1311 (15.9) | 2342 (10.2) |  | 966 (11.7) | 2687 (11.7) |  |
|  | 2020 | 1366 (16.6) | 3045 (13.3) |  | 1166 (14.2) | 3245 (14.2) |  |
|  | 2021 | 2171 (26.4) | 5687 (24.8) |  | 2077 (25.3) | 5782 (25.3) |  |
|  | 2022 | 2616 (31.8) | 9201 (40.2) |  | 3123 (38.0) | 8694 (38.0) |  |
|  | 2023 | 0 | 10 (<0.1) |  | 0 | 10 (<0.1) |  |
| Months of SGLT2i use in the study period before the index date, mean (SD) |  | 18.08 (15.47) | 8.42 (13.44) | **0.667** | 10.98 (14.69) | 10.98 (14.62) | <0.001 |
| Months of SGLT2i use in the whole database before the index date, mean (SD) |  | 25.07 (21.08) | 12.12 (18.31) | **0.656** | 15.54 (19.96) | 15.54 (19.91) | <0.001 |
| Months of T2D diagnosis between the first observed T2D diagnosis in the whole dataset available and the index date, mean (SD) |  | 51.37 (19.63) | 49.45 (22.51) | **0.091** | 49.96 (21.82) | 49.96 (21.80) | <0.001 |
| Baseline HbA_1c_, %, mean (SD) |  | 8.56 (1.59) | 8.00 (1.71) | **0.344** | 8.15 (1.70) | 8.15 (1.69) | <0.001 |
| Baseline HbA_1c_, categorical, n (%) | <7% | 844 (10.3) | 6316 (27.6) | **0.511** | 1815 (22.1) | 5357 (23.4) | 0.070 |
|  | 7% to <8% | 2241 (27.3) | 6973 (30.5) |  | 2467 (30.0) | 6955 (30.4) |  |
|  | 8% to <9% | 2454 (29.9) | 4465 (19.5) |  | 1936 (23.5) | 4746 (20.7) |  |
|  | 9% to <10% | 1304 (15.9) | 2263 (9.9) |  | 906 (11.0) | 2597 (11.3) |  |
|  | ≥10% | 1377 (16.8) | 2874 (12.6) |  | 1097 (13.3) | 3237 (14.1) |  |
| Baseline BMI, kg/m^2^, mean (SD) |  | 34.31 (6.80) | 31.73 (6.54) | **0.387** | 32.41 (6.72) | 32.41 (6.71) | <0.001 |
| Baseline BMI, categorical, n (%) | Normal or underweight | 398 (4.8) | 2578 (11.3) | **0.378** | 753 (9.2) | 2236 (9.8) | 0.037 |
|  | Overweight | 1726 (21.0) | 6487 (28.3) |  | 2240 (27.3) | 5968 (26.1) |  |
|  | Obesity class 1 | 2527 (30.7) | 7186 (31.4) |  | 2503 (30.5) | 7187 (31.4) |  |
|  | Obesity class 2 | 2004 (24.4) | 4225 (18.5) |  | 1641 (20.0) | 4593 (20.1) |  |
|  | Obesity class 3 | 1565 (19.0) | 2415 (10.5) |  | 1083 (13.2) | 2908 (12.7) |  |
| Baseline weight, kg, mean (SD) |  | 100.64 (23.05) | 91.70 (22.46) | **0.393** | 94.11 (22.90) | 94.03 (22.90) | 0.004 |
| Prescriber specialty for index drug, n (%) | Primary | 5891 (71.7) | 18 202 (79.5) | **0.371** | 6365 (77.4) | 17 728 (77.4) | 0.001 |
|  | Endocrinology | 1719 (20.9) | 1987 (8.7) |  | 978 (11.9) | 2727 (11.9) |  |
|  | Other | 296 (3.6) | 1604 (7.0) |  | 501 (6.1) | 1398 (6.1) |  |
|  | Unknown | 314 (3.8) | 1098 (4.8) |  | 375 (4.6) | 1039 (4.5) |  |
| Index drug, n (%) | Semaglutide OW T2D | 2697 (32.8) | - | - | 2546 (31.0) | - | - |
|  | Oral semaglutide | 1039 (12.6) | - |  | 1073 (13.0) | - |  |
|  | Tirzepatide T2D | 140 (1.7) | - |  | 140 (1.7) | - |  |
|  | Dulaglutide | 3991 (48.6) | - |  | 4106 (49.9) | - |  |
|  | Exenatide ER | 337 (4.1) | - |  | 340 (4.1) | - |  |
|  | Multiple | 16 (0.2) | - |  | 16 (0.2) | - |  |
| Baseline SGLT2i use and adherence, n (%) | <1 y SGLT2i use | 2938 (35.7) | 16 017 (70.0) | **0.747** | 5006 (60.9) | 13 947 (60.9) | 0.001 |
|  | SGLT2i PDC <50% | 213 (2.6) | 604 (2.6) |  | 216 (2.6) | 601 (2.6) |  |
|  | SGLT2i PDC <80% | 211 (2.6) | 418 (1.8) |  | 167 (2.0) | 463 (2.0) |  |
|  | SGLT2i PDC ≥80% | 4858 (59.1) | 5852 (25.6) |  | 2831 (34.4) | 7880 (34.4) |  |
| Number of GLTs used, n (%) | 0 | 427 (5.2) | 2058 (9.0) | **0.294** | 656 (8.0) | 1828 (8.0) | <0.001 |
|  | 1 | 2486 (30.2) | 9079 (39.7) |  | 3056 (37.2) | 8510 (37.2) |  |
|  | 2 | 3044 (37.0) | 7464 (32.6) |  | 2778 (33.8) | 7732 (33.8) |  |
|  | 3+ | 2263 (27.5) | 4290 (18.7) |  | 1731 (21.1) | 4821 (21.1) |  |
| Baseline GLT use, n (%) | Metformin | 6606 (80.4) | 17 634 (77.0) | 0.081 | 6405 (77.9) | 17 835 (77.9) | <0.001 |
|  | Sulfonylurea | 2605 (31.7) | 6451 (28.2) | 0.077 | 2393 (29.1) | 6663 (29.1) | <0.001 |
|  | DPP-4i | 2884 (35.1) | 5765 (25.2) | **0.217** | 2286 (27.8) | 6363 (27.8) | <0.001 |
|  | TZD | 717 (8.7) | 1522 (6.6) | 0.078 | 628 (7.6) | 1638 (7.2) | 0.019 |
|  | Basal insulin | 1979 (24.1) | 4130 (18.0) | **0.148** | 1614 (19.6) | 4495 (19.6) | <0.001 |
|  | Other insulin | 997 (12.1) | 2052 (9.0) | **0.103** | 806 (9.8) | 2244 (9.8) | <0.001 |
|  | AGI | 53 (0.6) | 111 (0.5) | 0.021 | 65 (0.8) | 117 (0.5) | 0.034 |
|  | MEG | 110 (1.3) | 205 (0.9) | 0.042 | 86 (1.0) | 221 (1.0) | 0.008 |
| CCI score excluding diabetes, mean (SD) |  | 0.98 (1.44) | 1.25 (1.67) | **0.173** | 1.20 (1.65) | 1.18 (1.62) | 0.014 |
| CCI score excluding diabetes, categorical, n (%) | 0-1 | 6234 (75.8) | 15 755 (68.8) | **0.170** | 5809 (70.7) | 16 179 (70.7) | 0.001 |
|  | 2 | 942 (11.5) | 3098 (13.5) |  | 1067 (13.0) | 2972 (13.0) |  |
|  | 3 | 501 (6.1) | 1762 (7.7) |  | 598 (7.3) | 1665 (7.3) |  |
|  | 4 | 283 (3.4) | 1044 (4.6) |  | 351 (4.3) | 976 (4.3) |  |
|  | 5 | 121 (1.5) | 529 (2.3) |  | 172 (2.1) | 479 (2.1) |  |
|  | 6+ | 139 (1.7) | 703 (3.1) |  | 223 (2.7) | 620 (2.7) |  |
| DCSI score, mean (SD) |  | 1.50 (1.76) | 1.75 (1.91) | **0.134** | 1.68 (1.87) | 1.68 (1.88) | 0.002 |
| DCSI score, categorical, n (%) | 0 | 3198 (38.9) | 8046 (35.1) | **0.142** | 2971 (36.1) | 8274 (36.1) | <0.001 |
|  | 1 | 1868 (22.7) | 4728 (20.7) |  | 1743 (21.2) | 4853 (21.2) |  |
|  | 2 | 1245 (15.1) | 3554 (15.5) |  | 1268 (15.4) | 3531 (15.4) |  |
|  | 3 | 835 (10.2) | 2544 (11.1) |  | 893 (10.9) | 2487 (10.9) |  |
|  | 4 | 462 (5.6) | 1725 (7.5) |  | 578 (7.0) | 1609 (7.0) |  |
|  | 5 | 281 (3.4) | 1082 (4.7) |  | 361 (4.4) | 1003 (4.4) |  |
|  | 6+ | 331 (4.0) | 1212 (5.3) |  | 407 (5.0) | 1135 (5.0) |  |
| Comorbidities, n (%) | Hypertension | 6794 (82.7) | 18 832 (82.3) | 0.010 | 6876 (83.6) | 18 747 (81.9) | 0.046 |
|  | Hyperlipidemia | 7075 (86.1) | 19 334 (84.5) | 0.045 | 7029 (85.5) | 19 346 (84.5) | 0.028 |
|  | Atrial fibrillation and flutter | 540 (6.6) | 2169 (9.5) | **0.107** | 726 (8.8) | 1963 (8.6) | 0.009 |
|  | HF | 646 (7.9) | 2771 (12.1) | **0.142** | 876 (10.7) | 2483 (10.8) | - |
|  | Depression | 1509 (18.4) | 3655 (16.0) | 0.063 | 1365 (16.6) | 3800 (16.6) | <0.001 |
|  | Anxiety | 1290 (15.7) | 3145 (13.7) | 0.055 | 1223 (14.9) | 3255 (14.2) | 0.019 |
|  | Smoking | 906 (11.0) | 2587 (11.3) | 0.009 | 990 (12.0) | 2579 (11.3) | 0.024 |
|  | Acute/chronic pancreatitis | 40 (0.5) | 309 (1.3) | 0.091 | 91 (1.1) | 257 (1.1) | 0.001 |
|  | MI | 539 (6.6) | 2067 (9.0) | 0.092 | 658 (8.0) | 1950 (8.5) | 0.019 |
|  | Ischemic stroke | 423 (5.1) | 1624 (7.1) | 0.081 | 542 (6.6) | 1539 (6.7) | 0.005 |
|  | PAD | 1353 (16.5) | 4968 (21.7) | **0.134** | 1657 (20.2) | 4704 (20.5) | 0.010 |
|  | TIA | 326 (4.0) | 1142 (5.0) | 0.049 | 417 (5.1) | 1103 (4.8) | 0.012 |
|  | ACS | 924 (11.2) | 3420 (14.9) | **0.110** | 1199 (14.6) | 3210 (14.0) | 0.016 |
|  | Other CHD | 2188 (26.6) | 7109 (31.1) | 0.098 | 2565 (31.2) | 6799 (29.7) | 0.033 |
|  | Obesity | 4374 (53.2) | 9308 (40.7) | **0.253** | 3615 (44.0) | 10 067 (44.0) | <0.001 |
|  | Anemia | 1138 (13.8) | 4106 (17.9) | **0.112** | 1463 (17.8) | 3787 (16.5) | 0.033 |
|  | Arrhythmia | 1536 (18.7) | 5397 (23.6) | **0.120** | 1808 (22.0) | 5067 (22.1) | 0.003 |
|  | Asthma | 1967 (23.9) | 4920 (21.5) | 0.058 | 1884 (22.9) | 5036 (22.0) | 0.022 |
|  | GERD | 1873 (22.8) | 5382 (23.5) | 0.017 | 1957 (23.8) | 5271 (23.0) | 0.018 |
|  | Musculoskeletal pain | 3542 (43.1) | 9944 (43.4) | 0.007 | 3605 (43.9) | 9927 (43.4) | 0.010 |
|  | MASH | 803 (9.8) | 2043 (8.9) | 0.029 | 923 (11.2) | 2075 (9.1) | 0.072 |
| Diseases/conditions related to weight changes, n (%) | Cancer | 1071 (13.0) | 3392 (14.8) | 0.052 | 1254 (15.3) | 3274 (14.3) | 0.027 |
|  | Cirrhosis | 113 (1.4) | 377 (1.6) | 0.022 | 133 (1.6) | 363 (1.6) | 0.002 |
|  | CKD | 15 (0.2) | 80 (0.3) | 0.032 | 28 (0.3) | 71 (0.3) | 0.004 |
|  | Eating disorders | 55 (0.7) | 190 (0.8) | 0.019 | 94 (1.1) | 171 (0.7) | 0.041 |
|  | Feeding disorders | 2 (<0.1) | 12 (0.1) | 0.014 | 5 (0.1) | 12 (0.1) | 0.004 |
|  | HIV | 74 (0.9) | 124 (0.5) | 0.042 | 99 (1.2) | 125 (0.5) | 0.071 |
| Procedures, n (%) | PCI | 43 (0.5) | 185 (0.8) | 0.035 | 42 (0.5) | 161 (0.7) | 0.025 |
|  | CABG | 30 (0.4) | 114 (0.5) | 0.020 | 36 (0.4) | 106 (0.5) | 0.004 |
|  | Major amputation | 13 (0.2) | 36 (0.2) | <0.001 | 9 (0.1) | 35 (0.2) | 0.014 |
|  | Dialysis | 329 (4.0) | 1329 (5.8) | 0.084 | 380 (4.6) | 1195 (5.2) | 0.027 |
|  | Percutaneous intervention-carotid | 17 (0.2) | 56 (0.2) | 0.008 | 18 (0.2) | 58 (0.3) | 0.007 |
|  | Percutaneous intervention-peripheral arteries | 33 (0.4) | 124 (0.5) | 0.020 | 31 (0.4) | 120 (0.5) | 0.023 |
|  | Thrombectomy/ thrombolysis | 20 (0.2) | 140 (0.6) | 0.056 | 23 (0.3) | 127 (0.6) | 0.043 |
| Number of OP visits at baseline, mean (SD) |  | 25.48 (35.77) | 25.97 (36.20) | 0.014 | 25.84 (32.32) | 25.84 (37.14) | <0.001 |
| Number of IP visits at baseline, mean (SD) |  | 0.13 (0.51) | 0.19 (0.69) | 0.103 | 0.18 (0.68) | 0.18 (0.65) | 0.001 |
| Number of T2D-related OP visits at baseline, mean (SD) |  | 7.69 (14.77) | 7.23 (14.26) | 0.032 | 7.38 (12.75) | 7.38 (15.21) | <0.001 |
| Number of T2D-related IP visits at baseline, mean (SD) |  | 0.09 (0.39) | 0.13 (0.50) | 0.071 | 0.11 (0.43) | 0.12 (0.48) | 0.016 |
| Number of OP visits 60 days before the index, mean (SD) |  | 4.92 (6.71) | 5.12 (6.87) | 0.029 | 5.07 (5.99) | 5.07 (7.07) | <0.001 |
| Number of IP visits 60 days before the index, mean (SD) |  | 0.02 (0.17) | 0.05 (0.25) | 0.099 | 0.04 (0.21) | 0.04 (0.24) | 0.011 |
| Number of T2D-related OP visits 60 days before the index, mean (SD) |  | 1.80 (2.92) | 1.67 (2.87) | 0.046 | 1.80 (2.56) | 1.66 (3.08) | 0.049 |
| Number of T2D-related IP visits 60 days before the index, mean (SD) |  | 0.02 (0.15) | 0.03 (0.20) | 0.066 | 0.03 (0.18) | 0.03 (0.19) | 0.003 |
| Medications, n (%) | Anticoagulants | 608 (7.4) | 2199 (9.6) | 0.079 | 709 (8.6) | 2032 (8.9) | 0.009 |
|  | Antidepressants | 2476 (30.1) | 5869 (25.6) | 0.100 | 2349 (28.6) | 6042 (26.4) | 0.049 |
|  | Antihyperlipidemic agents | 7171 (87.2) | 19 590 (85.6) | 0.048 | 6990 (85.0) | 19 599 (85.6) | 0.016 |
|  | Antihypertensive agents | 7096 (86.3) | 19 464 (85.0) | 0.037 | 7044 (85.7) | 19 424 (84.9) | 0.024 |
|  | Antiplatelets | 640 (7.8) | 2161 (9.4) | 0.059 | 714 (8.7) | 2085 (9.1) | 0.015 |
|  | ACE | 3818 (46.4) | 10 011 (43.7) | 0.055 | 3529 (42.9) | 10 189 (44.5) | 0.032 |
|  | ARB | 2768 (33.7) | 7867 (34.4) | 0.015 | 2869 (34.9) | 7642 (33.4) | 0.032 |
|  | Beta blocker | 2925 (35.6) | 8839 (38.6) | 0.063 | 3166 (38.5) | 8562 (37.4) | 0.023 |
|  | Loop diuretics | 854 (10.4) | 3087 (13.5) | 0.096 | 1128 (13.7) | 2844 (12.4) | 0.039 |
|  | MRA | 428 (5.2) | 1383 (6.0) | 0.036 | 456 (5.5) | 1255 (5.5) | 0.003 |
|  | Nitrates | 314 (3.8) | 1180 (5.2) | 0.065 | 362 (4.4) | 1100 (4.8) | 0.019 |
|  | Other HF medications | 175 (2.1) | 806 (3.5) | 0.084 | 234 (2.8) | 710 (3.1) | 0.015 |
|  | Other diuretics | 2376 (28.9) | 6171 (27.0) | 0.043 | 2392 (29.1) | 6129 (26.8) | 0.052 |
|  | Medication with weight gain/loss | 5672 (69.0) | 14 965 (65.4) | 0.077 | 5583 (67.9) | 15 053 (65.8) | 0.046 |

ACE indicates angiotensin-converting enzyme; ACS, acute coronary syndrome; AGI, alpha-glucosidase inhibitor; ARB, angiotensin receptor blocker; BMI, body mass index; CABG, coronary artery bypass grafting; CCI, Charlson Comorbidity Index; CHD, coronary heart disease; CKD, chronic kidney disease; DCSI, Diabetes Complications Severity Index; DPP-4i, dipeptidyl peptidase-4 inhibitor; ER, extended release; GERD, gastroesophageal reflux disease; GLP-1RA, glucagonlike peptide-1 receptor agonist; GLT, glucose-lowering therapy; HbA_1c_, glycated hemoglobin; HF, heart failure; IP, inpatient; MASH, metabolic dysfunction-associated steatohepatitis; MEG, meglitinide; MI, myocardial infarction; MRA, mineralocorticoid receptor agonist; OP, outpatient; OW, once weekly; PAD, peripheral artery disease; PCI, percutaneous coronary intervention; PDC, proportion of days covered; SGLT2i, sodium-glucose cotransporter 2 inhibitor; SMD, standardized mean difference; T2D, type 2 diabetes; TIA, transient ischemic attack; TZD, thiazolidinedione.

Bold font indicates statistical significance (SMD ≥0.1).

**Supplementary Table 4.** Unweighted and Weighted Baseline Characteristics of the T2D With CKD Cohort

|  |  | **Unweighted** | | | **Weighted** | | |
| --- | --- | --- | --- | --- | --- | --- | --- |
| **Characteristic** |  | **SGLT2i +  GLP-1RA** | **SGLT2i** | **SMD** | **SGLT2i + GLP-1RA** | **SGLT2i** | **SMD** |
| N |  | 3822 | 12578 |  | 3822 | 12578 |  |
| Age, y, mean (SD) |  | 62.39 (9.20) | 67.46 (10.38) | **0.517** | 66.28 (10.31) | 66.28 (10.34) | <0.001 |
| Age group, y, n (%) | 18-44 | 106 (2.8) | 202 (1.6) | **0.511** | 63 (1.6) | 242 (1.9) | 0.040 |
|  | 45-64 | 2369 (62.0) | 5146 (40.9) |  | 1789 (46.8) | 5754 (45.7) |  |
|  | 65-79 | 1202 (31.4) | 5509 (43.8) |  | 1571 (41.1) | 5140 (40.9) |  |
|  | 80+ | 145 (3.8) | 1721 (13.7) |  | 400 (10.5) | 1443 (11.5) |  |
| Sex, n (%) | Male | 1535 (40.2) | 5057 (40.2) | 0.017 | 2193 (57.4) | 7284 (57.9) | 0.017 |
|  | Female | 2221 (58.1) | 7275 (57.8) |  | 1566 (41.0) | 5067 (40.3) |  |
|  | Unknown | 66 (1.7) | 246 (2.0) |  | 64 (1.7) | 227 (1.8) |  |
| Insurance type, n (%) | Commercial | 1952 (51.1) | 4155 (33.0) | **0.413** | 1423 (37.2) | 4684 (37.2) | <0.001 |
|  | Medicare | 1320 (34.5) | 6770 (53.8) |  | 1885 (49.3) | 6205 (49.3) |  |
|  | Medicaid | 547 (14.3) | 1630 (13.0) |  | 507 (13.3) | 1670 (13.3) |  |
|  | Unknown | 3 (0.1) | 23 (0.2) |  | 6 (0.2) | 20 (0.2) |  |
| Geographic region, n (%) | Northeast | 1073 (28.1) | 3929 (31.2) | **0.118** | 1166 (30.5) | 3836 (30.5) | 0.002 |
|  | South | 1445 (37.8) | 4073 (32.4) |  | 1286 (33.6) | 4232 (33.6) |  |
|  | Midwest | 341 (8.9) | 1251 (9.9) |  | 371 (9.7) | 1221 (9.7) |  |
|  | West | 962 (25.2) | 3316 (26.4) |  | 997 (26.1) | 3281 (26.1) |  |
|  | Unknown | 1 (0.0) | 9 (0.1) |  | 2 (0.1) | 8 (0.1) |  |
| Race/ethnicity, n (%) | White | 1841 (48.2) | 5853 (46.5) | **0.170** | 1793 (46.9) | 5901 (46.9) | <0.001 |
|  | Black | 511 (13.4) | 1938 (15.4) |  | 571 (14.9) | 1878 (14.9) |  |
|  | Hispanic | 666 (17.4) | 2206 (17.5) |  | 669 (17.5) | 2202 (17.5) |  |
|  | Asian | 279 (7.3) | 1333 (10.6) |  | 376 (9.8) | 1236 (9.8) |  |
|  | Other | 150 (3.9) | 401 (3.2) |  | 128 (3.4) | 423 (3.4) |  |
|  | Unknown | 375 (9.8) | 847 (6.7) |  | 285 (7.5) | 937 (7.5) |  |
| Index year, n (%) | 2018 | 290 (7.6) | 1181 (9.4) | **0.295** | 328 (8.6) | 1137 (9.0) | 0.091 |
|  | 2019 | 544 (14.2) | 1045 (8.3) |  | 434 (11.3) | 1174 (9.3) |  |
|  | 2020 | 644 (16.8) | 1536 (12.2) |  | 458 (12.0) | 1715 (13.6) |  |
|  | 2021 | 1017 (26.6) | 3034 (24.1) |  | 924 (24.2) | 3104 (24.7) |  |
|  | 2022 | 1327 (34.7) | 5768 (45.9) |  | 1678 (43.9) | 5437 (43.2) |  |
|  | 2023 | - | 14 (0.1) |  | - | 11.7 (0.1) |  |
| Months of SGLT2i use in the study period before the index date, mean (SD) |  | 16.06 (14.95) | 7.71 (12.65) | **0.604** | 9.65 (13.77) | 9.65 (13.66) | <0.001 |
| Months of SGLT2i use in the whole database before the index date, mean (SD) |  | 22.04 (20.75) | 10.95 (17.55) | **0.577** | 13.54 (18.98) | 13.54 (18.93) | <0.001 |
| Months of T2D diagnosis between the first observed T2D diagnosis in the whole dataset available and the index date, mean (SD) |  | 53.91 (19.19) | 53.43 (22.34) | 0.023 | 53.54 (21.63) | 53.54 (21.64) | <0.001 |
| Months of CKD diagnosis between the first observed CKD diagnosis in the whole dataset available and the index date, mean (SD) |  | 11.75 (15.30) | 14.66 (18.72) | **0.170** | 12.86 (16.68) | 14.14 (18.07) | 0.074 |
| CKD stage, n (%) | 2 | 2679 (70.1) | 7784 (61.9) | **0.177** | 2406 (62.9) | 8068 (64.1) | 0.038 |
|  | 3 | 1057 (27.7) | 4373 (34.8) |  | 1278 (33.4) | 4131 (32.8) |  |
|  | 4 | 78 (2.0) | 397 (3.2) |  | 128 (3.3) | 356 (2.8) |  |
|  | 5 | 8 (0.2) | 24 (0.2) |  | 10 (0.3) | 23 (0.2) |  |
| Baseline eGFR, mL/min/1.73m^2^, mean (SD) |  | 67.85 (16.79) | 64.55 (17.66) | **0.192** | 65.32 (17.50) | 65.32 (17.52) | <0.001 |
| Baseline HbA_1c_, %, mean (SD) |  | 8.44 (1.55) | 7.68 (1.60) | **0.481** | 7.92 (1.58) | 7.87 (1.65) | 0.034 |
| Baseline HbA_1c_, categorical, n (%) | <7% | 436 (11.4) | 3684 (29.3) | **0.591** | 960 (25.1) | 3160 (25.1) | <0.001 |
|  | 7% to <8% | 935 (24.5) | 3156 (25.1) |  | 954 (24.9) | 3138 (24.9) |  |
|  | 8% to <9% | 996 (26.1) | 1794 (14.3) |  | 650 (17.0) | 2140 (17.0) |  |
|  | 9% to <10% | 499 (13.1) | 904 (7.2) |  | 327 (8.6) | 1076 (8.6) |  |
|  | ≥10% | 531 (13.9) | 942 (7.5) |  | 343 (9.0) | 1130 (9.0) |  |
|  | Unknown | 425 (11.1) | 2098 (16.7) |  | 588 (15.4) | 1935 (15.4) |  |
| Baseline BMI, kg/m^2^, mean (SD) |  | 33.76 (6.30) | 31.28 (6.29) | **0.394** | 32.06 (6.50) | 31.84 (6.34) | 0.034 |
| Baseline BMI, categorical, n (%) | Normal or underweight | 114 (3.0) | 961 (7.6) | **0.315** | 251 (6.6) | 825 (6.6) | <0.001 |
|  | Overweight | 553 (14.5) | 2335 (18.6) |  | 673 (17.6) | 2215 (17.6) |  |
|  | Obesity class 1 | 763 (20.0) | 2392 (19.0) |  | 735 (19.2) | 2420(19.2) |  |
|  | Obesity class 2 | 596 (15.6) | 1356 (10.8) |  | 455 (11.9) | 1497 (11.9) |  |
|  | Obesity class 3 | 466 (12.2) | 897 (7.1) |  | 318 (8.3) | 1045 (8.3) |  |
|  | Unknown | 1330 (34.8) | 4637 (36.9) |  | 1390 (36.4) | 4577 (36.4) |  |
| Patients with weight measurements, n (%) |  | 1701 (44.5) | 5452 (43.3) | 0.023 | 1627 (42.6) | 5458 (43.4) | 0.017 |
| Baseline weight, kg, mean (SD) |  | 99.50 (22.97) | 90.54 (22.22) | **0.396** | 93.26 (23.79) | 92.34 (22.55) | 0.040 |
| Prescriber specialty for index drug, n (%) | Primary | 2494 (65.3) | 8947 (71.1) | **0.483** | 2666 (69.8) | 8775 (69.8) | <0.001 |
|  | Endocrinology | 995 (26.0) | 1318 (10.5) |  | 539 (14.1) | 1774 (14.1) |  |
|  | Other | 211 (5.5) | 1830 (14.5) |  | 476 (12.4) | 1565 (12.4) |  |
|  | Unknown | 122 (3.2) | 483 (3.8) |  | 141 (3.7) | 464 (3.7) |  |
| Index drug, n (%) | Semaglutide OW T2D | 1300 (34.0) | - | - | 1300 (34.0) | - | - |
|  | Oral semaglutide | 508 (13.3) | - |  | 479 (12.5) | - |  |
|  | Tirzepatide T2D | 74 (1.9) | - |  | 78 (2.0) | - |  |
|  | Dulaglutide | 1785 (46.7) | - |  | 1813 (47.4) | - |  |
|  | Exenatide ER | 147 (3.8) | - |  | 145 (3.8) | - |  |
|  | Multiple | 8 (0.2) | - |  | 7 (0.2) | - |  |
| Baseline SGLT2i use and adherence, n (%) | <1 y SGLT2i use | 1669 (43.7) | 9242 (73.5) | **0.649** | 2543 (66.5) | 8368 (66.5) | 0.001 |
|  | SGLT2i PDC <50% | 111 (2.9) | 319 (2.5) |  | 100 (2.6) | 330 (2.6) |  |
|  | SGLT2i PDC <80% | 81 (2.1) | 210 (1.7) |  | 68 (1.8) | 223 (1.8) |  |
|  | SGLT2i PDC ≥80% | 1961 (51.3) | 2807 (22.3) |  | 1111 (29.1) | 3656 (29.1) |  |
| Number of GLTs used n (%) | 0 | 208 (5.4) | 1667 (13.3) | **0.397** | 437 (11.4) | 1438 (11.4) | <0.001 |
|  | 1 | 1004 (26.3) | 4361 (34.7) |  | 1251 (32.7) | 4115 (32.7) |  |
|  | 2 | 1322 (34.6) | 3979 (31.6) |  | 1235 (32.3) | 4066 (32.3) |  |
|  | 3+ | 1288 (33.7) | 2571 (20.4) |  | 899 (23.5) | 2960 (23.5) |  |
| Baseline GLT use, n (%) | Metformin | 2897 (75.8) | 8536 (67.9) | **0.177** | 2665 (69.7) | 8769 (69.7) | <0.001 |
|  | Sulfonylurea | 1363 (35.7) | 3685 (29.3) | **0.136** | 1176 (30.8) | 3872 (30.8) | <0.001 |
|  | DPP-4i | 1444 (37.8) | 3364 (26.7) | **0.238** | 1121 (29.3) | 3688 (29.3) | <0.001 |
|  | TZD | 366 (9.6) | 874 (6.9) | 0.096 | 314 (8.2) | 913 (7.3) | 0.036 |
|  | Basal insulin | 1146 (30.0) | 2555 (20.3) | **0.224** | 862 (22.6) | 2839 (22.6) | <0.001 |
|  | Another insulin | 620 (16.2) | 1388 (11.0) | **0.152** | 468 (12.2) | 1540 (12.2) | <0.001 |
|  | AGI | 34 (0.9) | 77 (0.6) | 0.032 | 32 (0.8) | 81 (0.6) | 0.022 |
|  | MEG | 77 (2.0) | 205 (1.6) | 0.029 | 78 (2.1) | 205 (1.6) | 0.031 |
| CCI score excluding diabetes, mean (SD) |  | 1.62 (1.72) | 2.11 (1.93) | **0.264** | 2.01 (2.02) | 1.99 (1.89) | 0.008 |
| CCI score excluding diabetes, categorical, n (%) | 0-1 | 2242 (58.7) | 5923 (47.1) | **0.262** | 1903 (49.8) | 6262 (49.8) | <0.001 |
|  | 2 | 666 (17.4) | 2352 (18.7) |  | 703 (18.4) | 2314 (18.4) |  |
|  | 3 | 412 (10.8) | 1739 (13.8) |  | 501 (13.1) | 1650 (13.1) |  |
|  | 4 | 259 (6.8) | 1169 (9.3) |  | 333 (8.7) | 1095 (8.7) |  |
|  | 5 | 114 (3.0) | 620 (4.9) |  | 171 (4.5) | 563 (4.5) |  |
|  | 6+ | 129 (3.4) | 775 (6.2) |  | 211 (5.5) | 694 (5.5) |  |
| DCSI score, mean (SD) |  | 2.26 (2.01) | 2.63 (2.06) | **0.182** | 2.55 (2.07) | 2.55 (2.06) | 0.001 |
| DCSI score, categorical, n (%) | 0 | 850 (22.2) | 2071 (16.5) | **0.199** | 681 (17.8) | 2240 (17.8) | <0.001 |
|  | 1 | 807 (21.1) | 2283 (18.2) |  | 720 (18.8) | 2370 (18.8) |  |
|  | 2 | 685 (17.9) | 2284 (18.2) |  | 692 (18.1) | 2277 (18.1) |  |
|  | 3 | 546 (14.3) | 2071 (16.5) |  | 610 (16.0) | 2007 (16.0) |  |
|  | 4 | 364 (9.5) | 1528 (12.1) |  | 441 (11.5) | 1451 (11.5) |  |
|  | 5 | 256 (6.7) | 1060 (8.4) |  | 307 (8.0) | 1010 (8.0) |  |
|  | 6+ | 314 (8.2) | 1281 (10.2) |  | 372 (9.7) | 1223 (9.7) |  |
| Comorbidities, n (%) | Hypertension | 3457 (90.5) | 11543 (91.8) | 0.046 | 3461 (90.5) | 11496 (91.4) | 0.030 |
|  | Hyperlipidemia | 3426 (89.6) | 11239 (89.4) | 0.009 | 3384 (88.5) | 11262 (89.5) | 0.032 |
|  | Atrial fibrillation and flutter | 394 (10.3) | 2018 (16.0) | **0.170** | 542 (14.2) | 1838 (14.6) | 0.012 |
|  | HF | 522 (13.7) | 2898 (23.0) | **0.244** | 703 (18.4) | 2614 (20.8) | 0.060 |
|  | Depression | 743 (19.4) | 2201 (17.5) | 0.050 | 686 (18.0) | 2258 (18.0) | <0.001 |
|  | Anxiety | 626 (16.4) | 1781 (14.2) | 0.062 | 573 (15.0) | 1822 (14.5) | 0.014 |
|  | Smoking | 408 (10.7) | 1415 (11.2) | 0.018 | 407 (10.6) | 1423 (11.3) | 0.022 |
|  | Acute/chronic pancreatitis | 39 (1.0) | 194 (1.5) | 0.046 | 54 (1.4) | 178 (1.4) | <0.001 |
|  | MI | 353 (9.2) | 1717 (13.7) | **0.139** | 376 (9.8) | 1623 (12.9) | 0.096 |
|  | Ischemic stroke | 288 (7.5) | 1397 (11.1) | **0.123** | 370 (9.7) | 1345 (10.7) | 0.034 |
|  | PAD | 1048 (27.4) | 4377 (34.8) | **0.160** | 1252 (32.8) | 4193 (33.3) | 0.012 |
|  | TIA | 220 (5.8) | 943 (7.5) | 0.070 | 255 (6.7) | 913 (7.3) | 0.023 |
|  | ACS | 661 (17.3) | 2920 (23.2) | **0.148** | 821 (21.5) | 2769 (22.0) | 0.013 |
|  | Other CHD | 1451 (38.0) | 5686 (45.2) | **0.147** | 1633 (42.7) | 5419 (43.1) | 0.007 |
|  | Obesity | 2134 (55.8) | 5459 (43.4) | **0.251** | 1770 (46.3) | 5824 (46.3) | <0.001 |
|  | Anemia | 877 (22.9) | 3712 (29.5) | **0.150** | 1085 (28.4) | 3486 (27.7) | 0.015 |
|  | Arrhythmia | 1043 (27.3) | 4472 (35.6) | **0.179** | 1226 (32.1) | 4228 (33.6) | 0.032 |
|  | Asthma | 956 (25.0) | 2844 (22.6) | 0.056 | 886 (23.2) | 2883 (22.9) | 0.006 |
|  | GERD | 1032 (27.0) | 3484 (27.7) | 0.016 | 1097 (28.7) | 3433 (27.3) | 0.031 |
|  | Musculoskeletal pain | 1875 (49.1) | 6100 (48.5) | 0.011 | 1923 (50.3) | 6097 (48.5) | 0.037 |
|  | MASH | 420 (11.0) | 1150 (9.1) | 0.061 | 387 (10.1) | 1190 (9.5) | 0.022 |
| Diseases/conditions related to weight changes, n (%) | Cancer | 679 (17.8) | 2760 (21.9) | **0.105** | 847 (22.2) | 2674 (21.3) | 0.022 |
|  | Cirrhosis | 87 (2.3) | 293 (2.3) | 0.004 | 112 (2.9) | 290 (2.3) | 0.038 |
|  | CKD | 4 (0.1) | 16 (0.1) | 0.007 | 5 (0.1) | 14 (0.1) | 0.009 |
|  | Eating Disorders | 30 (0.8) | 156 (1.2) | 0.045 | 46 (1.2) | 139 (1.1) | 0.010 |
|  | Feeding Disorders | 2 (0.1) | 19 (0.2) | 0.031 | 4 (0.1) | 17 (0.1) | 0.008 |
|  | HIV | 61 (1.6) | 151 (1.2) | 0.034 | 80 (2.1) | 151 (1.2) | 0.070 |
| Procedures, n (%) | PCI | 29 (0.8) | 177 (1.4) | 0.063 | 37 (1.0) | 165 (1.3) | 0.032 |
|  | CABG | 18 (0.5) | 114 (0.9) | 0.053 | 23 (0.6) | 106 (0.8) | 0.029 |
|  | Major amputation | 5 (0.1) | 30 (0.2) | 0.025 | 3 (0.1) | 32 (0.3) | 0.046 |
|  | Bariatric | 5 (0.1) | 35 (0.3) | 0.033 | 8 (0.2) | 35 (0.3) | 0.016 |
|  | Dialysis | - | - | <0.001 | - | - | <0.001 |
|  | Percutaneous intervention-carotid | 10 (0.3) | 52 (0.4) | 0.026 | 12 (0.3) | 52 (0.4) | 0.018 |
|  | Percutaneous intervention-peripheral arteries | 25 (0.7) | 114 (0.9) | 0.029 | 26 (0.7) | 107 (0.9) | 0.021 |
|  | Thrombectomy/ thrombolysis | 16 (0.4) | 110 (0.9) | 0.057 | 19 (0.5) | 101 (0.8) | 0.038 |
| Number of OP visits at baseline, mean (SD) |  | 31.53 (41.08) | 33.27 (43.05) | 0.041 | 32.86 (40.70) | 32.86 (42.96) | <0.001 |
| Number of IP visits at baseline, mean (SD) |  | 0.19 (0.57) | 0.32 (0.85) | **0.179** | 0.25 (0.75) | 0.30 (0.81) | 0.061 |
| Number of T2D-related OP visits at baseline, mean (SD) |  | 9.37 (17.04) | 8.75 (16.73) | 0.037 | 9.18 (18.74) | 9.00 (17.57) | 0.010 |
| Number of T2D-related IP visits at baseline, mean (SD) |  | 0.13 (0.45) | 0.19 (0.59) | **0.107** | 0.15 (0.57) | 0.18 (0.57) | 0.050 |
| Number of OP visits 60 days before the index, mean (SD) |  | 5.96 (7.52) | 6.35 (8.13) | 0.051 | 6.26 (7.52) | 6.26 (8.13) | <0.001 |
| Number of IP visits 60 days before the index, mean (SD) |  | 0.03 (0.19) | 0.08 (0.33) | **0.179** | 0.05 (0.23) | 0.07 (0.30) | 0.077 |
| Number of T2D-related OP visits 60 days before the index, mean (SD) |  | 2.07 (3.29) | 1.86 (3.38) | 0.065 | 2.05 (3.53) | 1.88 (3.50) | 0.048 |
| Number of T2D-related IP visits 60 days before the index, mean (SD) |  | 0.02 (0.16) | 0.05 (0.24) | **0.128** | 0.03 (0.18) | 0.04 (0.23) | 0.062 |
| Medications, n (%) | Anticoagulants | 412 (10.8) | 2018 (16.0) | **0.155** | 541 (14.2) | 1890 (15.0) | 0.025 |
|  | Antidepressants | 1202 (31.4) | 3317 (26.4) | **0.112** | 1169 (30.6) | 3388 (26.9) | 0.081 |
|  | Antihyperlipidemic agent | 3457 (90.5) | 11250 (89.4) | 0.034 | 3317 (86.8) | 11255 (89.5) | 0.084 |
|  | Antihypertensive agent | 3527 (92.3) | 11671 (92.8) | 0.019 | 3455 (90.4) | 11602 (92.2) | 0.066 |
|  | Antiplatelets | 429 (11.2) | 1754 (13.9) | 0.082 | 487 (12.7) | 1686 (13.4) | 0.020 |
|  | ACE | 1707 (44.7) | 5267 (41.9) | 0.056 | 1522 (39.8) | 5366 (42.7) | 0.058 |
|  | ARB | 1607 (42.0) | 5583 (44.4) | 0.047 | 1609 (42.1) | 5413 (43.0) | 0.019 |
|  | Beta blocker | 1822 (47.7) | 6760 (53.7) | **0.122** | 1881 (49.2) | 6529 (51.9) | 0.054 |
|  | Loop diuretics | 666 (17.4) | 3056 (24.3) | **0.170** | 779 (20.4) | 2820 (22.4) | 0.050 |
|  | MRA | 295 (7.7) | 1441 (11.5) | **0.127** | 311 (8.1) | 1329 (10.6) | 0.084 |
|  | Nitrates | 242 (6.3) | 1161 (9.2) | **0.108** | 291 (7.6) | 1082 (8.6) | 0.036 |
|  | Other HF medications | 143 (3.7) | 992 (7.9) | **0.178** | 182 (4.8) | 866 (6.9) | 0.091 |
|  | Other diuretics | 1371 (35.9) | 4060 (32.3) | 0.076 | 1359 (35.6) | 4058(32.3) | 0.070 |
|  | Medications with weight gain/loss | 2970 (77.7) | 9384 (74.6) | 0.073 | 2897 (75.8) | 9430 (75.0) | 0.019 |

ACE indicates angiotensin-converting enzyme; ACS, acute coronary syndrome; AGI, alpha-glucosidase inhibitor; ARB, angiotensin receptor blocker; BMI, body mass index; CABG, coronary artery bypass grafting; CCI, Charlson Comorbidity Index; CHD, coronary heart disease; CKD, chronic kidney disease; DCSI, Diabetes Complications Severity Index; DPP-4i, dipeptidyl peptidase-4 inhibitor; ER, extended release; GERD, gastroesophageal reflux disease; GLP-1RA, glucagonlike peptide-1 receptor agonist; GLT, glucose-lowering therapy; HbA_1c_, glycated hemoglobin; HF, heart failure; IP, inpatient; MASH, metabolic dysfunction-associated steatohepatitis; MEG, meglitinide; MI, myocardial infarction; MRA, mineralocorticoid receptor agonist; OP, outpatient; OW, once weekly; PAD, peripheral artery disease; PCI, percutaneous coronary intervention; PDC, proportion of days covered; SGLT2i, sodium-glucose cotransporter 2 inhibitor; SMD, standardized mean difference; T2D, type 2 diabetes; TIA, transient ischemic attack; TZD, thiazolidinedione.

Bold font indicates statistical significance (SMD ≥0.1).

**Supplementary Table 5**. Detailed Weighted Renal Outcomes for Drug Class and Individual Drug Combination Therapy With GLP-1RA and SGLT2i Compared With SGLT2i

|  | **6 mo** |  | **12 mo** |  | **18 mo** |  |
| --- | --- | --- | --- | --- | --- | --- |
|  | **GLP-1RA + SGLT2i** | **SGLT2i** | **GLP-1RA + SGLT2i** | **SGLT2i** | **GLP-1RA + SGLT2i** | **SGLT2i** |
| **GLP-1RA^a^ + SGLT2i vs SGLT2i** | | | | | | |
| n | 3023 | 9900 | 2879 | 8860 | 1928 | 6238 |
| Baseline eGFR, mL/min/1.73 m^2^, mean (SD) | 65.04 (17.75) | 64.79 (17.68) | 65.22 (17.66) | 65.08 (17.55) | 65.80 (16.73) | 66.36 (17.12) |
| Follow-up eGFR, mL/min/1.73 m^2^, mean (SD) | 65.56 (19.09) | 64.71 (19.11) | 66.37 (19.74) | 65.06 (19.31) | 66.78 (18.84) | 66.24 (19.20) |
| Difference (95% CI)  *P* value | 0.85 (-0.59, 2.29)  .246 | | 1.31 (-0.27, 2.89)  .105 | | 0.54 (-1.22, 2.29)  .548 | |
| eGFR change, mL/min/1.73 m^2^,  mean (SD) | 0.52 (11.33) | -0.08 (11.53) | 1.14 (12.94) | -0.03 (12.18) | 0.97 (12.85) | -0.12 (13.03) |
| Difference (95% CI)  *P* value | 0.60 (-0.19, 1.38)  .135 | | 1.17 (0.22, 2.13)  **.016** | | 1.09 (0.03, 2.15)  **.043** | |
| **Semaglutide OW + SGLT2i vs SGLT2i** | | | | | | |
| n | 1029 | 9898 | 984 | 8601 | 732 | 6566 |
| Baseline eGFR, mL/min/1.73 m^2^, mean (SD) | 67.99 (16.56) | 67.84 (16.54) | 68.41 (16.49) | 68.00 (16.41) | 69.00 (16.11) | 69.06 (16.10) |
| Follow-up eGFR, mL/min/1.73 m^2^, mean (SD) | 69.69 (17.69) | 68.20 (17.82) | 70.27 (17.50) | 68.01 (18.04) | 70.58 (17.65) | 69.41 (18.13) |
| Difference (95% CI)  *P* value | 1.49 (0.17, 2.81)  **.026** | | 2.26 (0.91, 3.61)  **.001** | | 1.16 (-0.46, 2.79)  .160 | |
| eGFR change, mL/min/1.73 m^2^,  mean (SD) | 1.70 (10.68) | 0.36 (11.74) | 1.86 (11.80) | 0.01 (12.41) | 1.58 (12.15) | 0.36 (13.37) |
| Difference (95% CI)  *P* value | 1.34 (0.51, 2.17)  **.002** | | 1.85 (0.89, 2.81)  **<.001** | | 1.22 (0.00, 2.44)  **.050** | |
| **Oral semaglutide + SGLT2i vs SGLT2i** | | | | | | |
| n | 405 | 9773 | 380 | 8470 | 277 | 6439 |
| Baseline eGFR, mL/min/1.73 m^2^, mean (SD) | 66.58 (17.89) | 66.35 (18.49) | 67.75 (17.97) | 66.56 (18.44) | 68.81 (16.65) | 68.25 (17.66) |
| Follow-up eGFR, mL/min/1.73 m^2^, mean (SD) | 67.62 (18.81) | 66.68 (19.36) | 69.43 (19.07) | 66.73 (19.48) | 71.17 (18.18) | 68.43 (19.21) |
| Difference (95% CI)  *P* value | 0.93 (-1.21, 3.08)  .394 | | 2.71 (0.44, 4.97)  **.019** | | 2.74 (0.20, 5.29)  **.035** | |
| eGFR change, mL/min/1.73 m^2^,  mean (SD) | 1.04 (10.32) | 0.34 (11.44) | 1.69 (11.38) | 0.16 (11.86) | 2.36 (12.08) | 0.17 (12.72) |
| Difference (95% CI)  *P* value | 0.70 (-0.49, 1.90)  .250 | | 1.52 (0.16, 2.89)  **.028** | | 2.19 (0.54, 3.84)  **.009** | |
| **Dulaglutide + SGLT2i vs SGLT2i** | | | | | | |
| n | 1405 | 9803 | 1326 | 8552 | 1036 | 6735 |
| Baseline eGFR, mL/min/1.73 m^2^, mean (SD) | 67.31 (16.85) | 67.09 (16.96) | 67.55 (16.84) | 67.20 (16.72) | 68.03 (16.45) | 68.24 (16.43) |
| Follow-up eGFR, mL/min/1.73 m^2^, mean (SD) | 68.07 (17.79) | 67.48 (18.33) | 69.54 (18.20) | 67.56 (18.33) | 68.79 (17.89) | 68.66 (18.65) |
| Difference (95% CI)  *P* value | 0.59 (-0.61, 1.80)  .335 | | 1.98 (0.71, 3.25)  **.002** | | 0.13 (-1.36, 1.62)  .864 | |
| eGFR change, mL/min/1.73 m^2^,  mean (SD) | 0.76 (11.14) | 0.39 (12.03) | 1.99 (12.42) | 0.36 (12.83) | 0.75 (12.76) | 0.41 (13.16) |
| Difference (95% CI)  *P* value | 0.38 (-0.42, 1.17)  .356 | | 1.63 (0.70, 2.56)  **<.001** | | 0.34 (-0.71, 1.39)  .524 | |

eGFR indicates estimated glomerular filtration rate; GLP-1RA, glucagonlike peptide-1 receptor agonist; OW, once weekly; SGLT2i, sodium-glucose cotransporter 2 inhibitor.

^a^GLP-1RAs included semaglutide OW T2D, oral semaglutide, dulaglutide, exenatide OW, and tirzepatide T2D.

Bold font indicates statistical significance (*P*≤.05).

**Supplementary Table 6.** CV Outcomes Stratified by Age Group

|  | **<65 y old** |  | | **≥65 y old** | |  |
| --- | --- | --- | --- | --- | --- | --- |
|  | **GLP-1RA^a^ + SGLT2i**  **n=20 101** | | **SGLT2i**  **n=75 575** | **GLP-1RA^a^ + SGLT2i**  **n=14 589** | **SGLT2i**  **n=54 645** | |
| **Ischemic stroke** | | | | | | |
| Event count, n (%) | 115.0 (0.6) | | 630.0 (0.8) | 90.8 (0.6) | 652.4 (1.2) | |
| Incidence rate (per 1000 PY) | 4.54 | | 6.83 | 5.12 | 10.47 | |
| HR (95% CI) | **0.67 (0.52, 0.86)** | | | **0.49 (0.35, 0.68)** | | |
| **Myocardial infarction** | | | | | | |
| Event count, n (%) | 145.4 (0.7) | | 896.7 (1.2) | 145.1 (1.0) | 765.5 (1.4) | |
| Incidence rate (per 1000 PY) | 5.75 | | 9.74 | 8.20 | 12.30 | |
| HR (95% CI) | **0.59 (0.48, 0.74)** | | | **0.67 (0.50, 0.90)** | | |
| **3-point MACE** | | | | | | |
| Event count, n (%) | 534.1 (2.7) | | 3638.0 (4.8) | 757.4 (5.2) | 4830.1 (8.8) | |
| Incidence rate (per 1000 PY) | 21.18 | | 39.64 | 42.90 | 78.04 | |
| HR (95% CI) | **0.53 (0.48, 0.60)** | | | **0.54 (0.47, 0.61)** | | |
| **5-point MACE** | | | | | | |
| Event count, n (%) | 691.6 (3.4) | | 4727.3 (6.3) | 935.6 (6.4) | 5830.2 (10.7) | |
| Incidence rate (per 1000 PY) | 27.53 | | 51.96 | 53.34 | 95.29 | |
| HR (95% CI) | **0.53 (0.48, 0.59)** | | | **0.55 (0.50, 0.62)** | | |

CV indicates cardiovascular; GLP-1RA, glucagonlike peptide-1 receptor agonist; HR, hazard ratio; MACE, major adverse cardiovascular event; PY, person-years; SGLT2i, sodium-glucose cotransporter 2 inhibitor.

^a^GLP-1RAs included semaglutide OW T2D, oral semaglutide, dulaglutide, exenatide OW, and tirzepatide T2D.

Bold font indicates statistical significance (*P*≤.05).

**Supplementary Table 7.** HbA_1c_ and Weight Outcomes Stratified by Age Group

|  | **<65 y old** | | | **≥65 y old** | | |  |
| --- | --- | --- | --- | --- | --- | --- | --- |
|  | **GLP-1RA^a^ + SGLT2i** | **SGLT2i** | **GLP-1RA^a^ + SGLT2i vs SGLT2i** | **GLP-1RA^a^ + SGLT2i** | **SGLT2i** | **GLP-1RA^a^ + SGLT2i vs SGLT2i** |  |
| **6 mo** | | | | | | | |
| **HbA_1c_ outcomes** | | | | | | | |
| n | 4188 | 11 154 |  | 2302 | 6400 |  |  |
| Baseline HbA_1c_, %, mean (SD) | 8.18 (1.73) | 8.22 (1.74) |  | 8.06 (1.59) | 7.94 (1.52) |  |  |
| Follow-up HbA_1c_, %, mean (SD) | 6.91 (1.27) | 7.48 (1.45) |  | 7.15 (1.26) | 7.49 (1.33) |  |  |
| Difference (95% CI) |  |  | **-0.58 (-0.64, -0.51)** |  |  | **-0.34 (-0.47, -0.22)** |  |
| HbA_1c_ change, %, mean (SD) | -1.27 (1.72) | -0.73 (1.73) |  | -0.91 (1.57) | -0.45 (1.42) |  |  |
| Difference (95% CI) |  |  | **-0.53 (-0.62, -0.45)** |  |  | **-0.46 (-0.61, -0.32)** |  |
| Follow-up HbA_1c_ <7%, n (%) | 2494.9 (59.6) | 4333.3 (38.8) |  | 1127.7 (49.0) | 2270.5 (35.5) |  |  |
| OR (95% CI) |  |  | **2.32 (2.10, 2.56)** |  |  | **1.75 (1.44, 2.11)** |  |
| Follow-up HbA_1c_ <8%, n (%) | 3530.3 (84.3) | 8104.2 (72.7) |  | 1845.8 (80.2) | 4677.8 (73.1) |  |  |
| OR (95% CI) |  |  | **2.02 (1.79, 2.28)** |  |  | **1.49 (1.21, 1.84)** |  |
| **Weight outcomes** | | | | | | | |
| n | 4575 | 12 225 |  | 2552 | 7129 |  |  |
| Baseline weight, kg, mean (SD) | 98.10 (23.15) | 97.16 (23.52) |  | 87.34 (20.92) | 88.31 (20.57) |  |  |
| Follow-up weight, kg, mean (SD) | 94.58 (22.76) | 95.60 (23.33) |  | 83.90 (20.42) | 86.70 (20.34) |  |  |
| Difference (95% CI) |  |  | -1.02 (-2.14, 0.09) |  |  | **-2.79 (-4.50, -1.09)** |  |
| Weight change, kg, mean (SD) | -3.52 (6.23) | -1.56 (5.12) |  | -3.44 (4.68) | -1.61 (5.02) |  |  |
| Difference (95% CI) |  |  | **-1.96 (-2.29, -1.64)** |  |  | **-1.83 (-2.27, -1.39)** |  |
| Weight change >5%, n (%) | 1649.5 (36.1) | 2186.8 (17.9) |  | 988.4 (38.7) | 1340.9 (18.8) |  |  |
| OR (95% CI) |  |  | **2.59 (2.33, 2.88)** |  |  | **2.73 (2.25, 3.30)** |  |
| Weight change >10%, n (%) | 498.5 (10.9) | 464.0 (3.8) |  | 283.9 (11.1) | 294.4 (4.1) |  |  |
| OR (95% CI) |  |  | **3.10 (2.57, 3.74)** |  |  | **2.91 (2.09, 4.05)** |  |
| Weight change >15%, n (%) | 128.5 (2.8) | 127.3 (1.0) |  | 66.2 (2.6) | 79.7 (1.1) |  |  |
| OR (95% CI) |  |  | **2.74 (1.92, 3.92)** |  |  | **2.36 (1.26, 4.42)** |  |
| Baseline BMI, kg/m^2^, mean (SD) | 33.46 (6.88) | 33.19 (6.89) |  | 30.67 (6.08) | 31.02 (6.17) |  |  |
| Follow-up BMI, kg/m^2^, mean (SD) | 32.24 (6.67) | 32.65 (6.82) |  | 29.45 (5.86) | 30.46 (6.13) |  |  |
| Difference (95% CI) |  |  | **-0.41 (-0.74, -0.09)** |  |  | **-1.01 (-1.49, -0.53)** |  |
| BMI change, kg/m^2^, mean (SD) | -1.22 (2.11) | -0.54 (1.74) |  | -1.23 (1.65) | -0.56 (1.77) |  |  |
| Difference (95% CI) |  |  | **-0.69 (-0.79, -0.58)** |  |  | -0.66 (-0.82, -0.51) |  |
| **12 mo** | | | | | | | |
| **HbA_1c_ outcomes** | | | | | | | |
| n | 3792 | 9936 |  | 2060 | 5815 |  |  |
| Baseline HbA_1c_, %, mean (SD) | 8.15 (1.70) | 8.20 (1.75) |  | 8.15 (1.54) | 7.93 (1.49) |  |  |
| Follow-up HbA_1c_, %, mean (SD) | 7.04 (1.41) | 7.54 (1.50) |  | 7.24 (1.26) | 7.47 (1.33) |  |  |
| Difference (95% CI) |  |  | **-0.50 (-0.57, -0.42)** |  |  | **-0.23 (-0.34, -0.12)** |  |
| HbA_1c_ change, %, mean (SD) | -1.11 (1.74) | -0.67 (1.76) |  | -0.91 (1.58) | -0.47 (1.49) |  |  |
| Difference (95% CI) |  |  | **-0.44 (-0.53, -0.35)** |  |  | **-0.44 (-0.59, -0.29)** |  |
| Follow-up HbA_1c_ <7%, n (%) | 2063.3 (54.4) | 3755.8 (37.8) |  | 928.4 (45.1) | 2138.8 (36.8) |  |  |
| OR (95% CI) |  |  | **1.96 (1.77, 2.18)** |  |  | **1.41 (1.16, 1.71)** |  |
| Follow-up HbA_1c_ <8%, n (%) | 3085.2 (81.4) | 7078.0 (71.2) |  | 1606.9 (78.0) | 4273.8 (73.5) |  |  |
| OR (95% CI) |  |  | **1.76 (1.56, 1.99)** |  |  | **1.28 (1.03, 1.59)** |  |
| **Weight outcomes** | | | | | | | |
| n | 4255 | 11 255 |  | 2385 | 6683 |  |  |
| Baseline weight, kg, mean (SD) | 98.27 (23.01) | 97.24 (23.56) |  | 87.31 (20.88) | 88.41 (20.65) |  |  |
| Follow-up weight, kg, mean (SD) | 94.13 (22.75) | 95.45 (23.26) |  | 82.95 (20.33) | 86.36 (20.42) |  |  |
| Difference (95% CI) |  |  | **-1.32 (-2.47, -0.17)** |  |  | **-3.41 (-5.14, -1.68)** |  |
| Weight change, kg, mean (SD) | -4.14 (7.88) | -1.79 (6.11) |  | -4.36 (6.54) | -2.05 (5.89) |  |  |
| Difference (95% CI) |  |  | **-2.35 (-2.78, -1.93)** |  |  | **-2.31 (-2.84, -1.78)** |  |
| Weight change >5%, n (%) | 1697.9 (39.9) | 2367.2 (21.0) |  | 1087.5 (45.6) | 1648.4 (24.7) |  |  |
| OR (95% CI) |  |  | **2.49 (2.24, 2.77)** |  |  | **2.56 (2.13, 3.08)** |  |
| Weight change >10%, n (%) | 663.9 (15.6) | 655.7 (5.8) |  | 391.4 (16.4) | 469.6 (7.0) |  |  |
| OR (95% CI) |  |  | **2.99 (2.56, 3.49)** |  |  | **2.60 (2.01, 3.35)** |  |
| Weight change >15%, n (%) | 259.3 (6.1) | 242.4 (2.2) |  | 163.0 (6.8) | 170.8 (2.6) |  |  |
| OR (95% CI) |  |  | **2.95 (2.30, 3.79)** |  |  | **2.80 (1.91, 4.10)** |  |
| Baseline BMI, kg/m^2^, mean (SD) | 33.50 (6.78) | 33.22 (6.90) |  | 30.65 (6.18) | 31.05 (6.17) |  |  |
| Follow-up BMI, kg/m^2^, mean (SD) | 32.06 (6.60) | 32.61 (6.80) |  | 29.10 (5.94) | 30.33 (6.13) |  |  |
| Difference (95% CI) |  |  | **-0.55 (-0.88, -0.22)** |  |  | **-1.23 (-1.72, -0.73)** |  |
| BMI change, kg/m^2^, mean (SD) | -1.44 (2.70) | -0.61 (2.10) |  | -1.55 (2.35) | -0.72 (2.07) |  |  |
| Difference (95% CI) |  |  | **-0.83 (-0.97, -0.68)** |  |  | **-0.83 (-1.03, -0.64)** |  |
| **18 mo** | | | | | | | |
| **HbA_1c_ outcomes** | | | | | | | |
| n | 2830 | 7496 |  | 1617 | 4239 |  |  |
| Baseline HbA_1c_, %, mean (SD) | 8.24 (1.69) | 8.20 (1.73) |  | 8.14 (1.63) | 7.97 (1.50) |  |  |
| Follow-up HbA_1c_, %, mean (SD) | 7.17 (1.42) | 7.61 (1.60) |  | 7.24 (1.35) | 7.48 (1.36) |  |  |
| Difference (95% CI) |  |  | **-0.45 (-0.53, -0.36)** |  |  | **-0.24 (-0.39, -0.09)** |  |
| HbA_1c_ change, %, mean (SD) | -1.07 (1.86) | -0.58 (1.84) |  | -0.90 (1.66) | -0.49 (1.55) |  |  |
| Difference (95% CI) |  |  | **-0.49 (-0.59, -0.38)** |  |  | **-0.41 (-0.59, -0.23)** |  |
| Follow-up HbA_1c_ <7%, n (%) | 1423.6 (50.3) | 2744.4 (36.6) |  | 757.7 (46.8) | 1548.0 (36.5) |  |  |
| OR (95% CI) |  |  | **1.75 (1.56, 1.97)** |  |  | **1.53 (1.21, 1.94)** |  |
| Follow-up HbA_1c_ <8%, n (%) | 2226.8 (78.7) | 5209.9 (69.5) |  | 1258.8 (77.8) | 3080.8 (72.7) |  |  |
| OR (95% CI) |  |  | **1.62 (1.42, 1.85)** |  |  | **1.32 (1.03, 1.70)** |  |
| **Weight outcomes** | | | | | | | |
| n | 3329 | 8599 |  | 1885 | 4958 |  |  |
| Baseline weight, kg, mean (SD) | 97.83 (22.82) | 97.10 (23.32) |  | 87.03 (20.85) | 88.71 (20.62) |  |  |
| Follow-up weight, kg, mean (SD) | 93.78 (22.75) | 94.97 (22.95) |  | 82.88 (20.17) | 86.36 (20.30) |  |  |
| Difference (95% CI) |  |  | -1.19 (-2.49, 0.11) |  |  | **-3.47 (-5.49, -1.46)** |  |
| Weight change, kg, mean (SD) | -4.05 (8.08) | -2.13 (6.73) |  | -4.15 (6.49) | -2.35 (6.29) |  |  |
| Difference (95% CI) |  |  | **-1.92 (-2.40, -1.44)** |  |  | **-1.80 (-2.42, -1.18)** |  |
| Weight change >5%, n (%) | 1373.1 (41.2) | 2137.2 (24.9) |  | 843.2 (44.7) | 1390.7 (28.1) |  |  |
| OR (95% CI) |  |  | **2.12 (1.88, 2.39)** |  |  | **2.08 (1.67, 2.57)** |  |
| Weight change >10%, n (%) | 578.9 (17.4) | 684.6 (8.0) |  | 371.1 (19.7) | 455.2 (9.2) |  |  |
| OR (95% CI) |  |  | **2.43 (2.05, 2.88)** |  |  | **2.43 (1.82, 3.23)** |  |
| Weight change >15%, n (%) | 202.9 (6.1) | 269.0 (3.1) |  | 123.1 (6.5) | 160.0 (3.2) |  |  |
| OR (95% CI) |  |  | **2.01 (1.54, 2.63)** |  |  | **2.10 (1.41, 3.12)** |  |
| Baseline BMI, kg/m^2^, mean (SD) | 33.31 (6.62) | 33.17 (6.80) |  | 30.51 (6.16) | 31.10 (6.15) |  |  |
| Follow-up BMI, kg/m^2^, mean (SD) | 31.90 (6.58) | 32.43 (6.67) | - | 29.02 (5.78) | 30.29 (6.11) | - |  |
| Difference (95% CI) |  |  | **-0.53 (-0.91, -0.15)** |  |  | **-1.27 (-1.83, -0.71)** |  |
| BMI change, kg/m^2^, mean (SD) | -1.40 (2.80) | -0.73 (2.34) |  | -1.49 (2.31) | -0.82 (2.21) |  |  |
| Difference (95% CI) |  |  | **-0.67 (-0.84, -0.50)** |  |  | **-0.68 (-0.91, -0.45)** |  |

BMI indicates body mass index; GLP-1RA, glucagonlike peptide-1 receptor agonist; HbA_1c_, glycated hemoglobin; OR, odds ratio; SGLT2i, sodium-glucose cotransporter 2 inhibitor.

^a^GLP-1RAs included semaglutide OW T2D, oral semaglutide, dulaglutide, exenatide OW, and tirzepatide T2D.

Bold font indicates statistical significance (*P*≤0.05).

**Supplementary Table 8.** Renal Outcomes Stratified by Age Group

|  | **<65 y old** | | | **≥65 y old** | | |  |
| --- | --- | --- | --- | --- | --- | --- | --- |
|  | **GLP-1RA^a^ + SGLT2i** | **SGLT2i** | **Difference (95% CI)** | **GLP-1RA^a^ + SGLT2i** | **SGLT2i** | **Difference (95% CI)** |  |
| **6 mo** | | | | | | | |
| n | 1416 | 4637 | - | 1607 | 5264 | - |  |
| Baseline eGFR, mL/min/1.73 m^2^, mean (SD) | 70.63 (15.79) | 69.95 (16.46) | - | 60.12 (17.93) | 60.24 (17.47) | - |  |
| Follow-up eGFR, mL/min/1.73 m^2^, mean (SD) | 71.78 (17.15) | 70.25 (18.13) | **1.53 (0.25, 2.81)** | 60.08 (19.04) | 59.82 (18.61) | 0.25 (-2.06, 2.56) |  |
| eGFR change, mL/min/1.73 m^2^, mean (SD) | 1.15 (11.25) | 0.30 (12.00) | 0.85 (-0.01, 1.71) | -0.04 (11.37) | -0.42 (11.08) | 0.38 (-0.88, 1.63) |  |
| **12 mo** | | | | | | | |
| n | 1382 | 4076 | - | 1497 | 4784 | - |  |
| Baseline eGFR, mL/min/1.73 m^2^, mean (SD) | 70.40 (15.89) | 70.28 (16.39) | - | 60.44 (17.87) | 60.66 (17.29) | - |  |
| Follow-up eGFR, mL/min/1.73 m^2^, mean (SD) | 72.88 (17.91) | 70.45 (18.75) | **2.43 (0.91, 3.95)** | 60.36 (19.44) | 60.46 (18.58) | -0.11 (-2.62, 2.41) |  |
| eGFR change, mL/min/1.73 m^2^, mean (SD) | 2.47 (12.62) | 0.17 (12.77) | **2.31 (1.07, 3.54)** | -0.08 (13.12) | -0.19 (11.66) | 0.11 (-1.29, 1.51) |  |
| **18 mo** | | | | | | | |
| n | 933 | 3006 | - | 995 | 3232 | - |  |
| Baseline eGFR, mL/min/1.73 m^2^, mean (SD) | 71.29 (14.79) | 71.35 (15.78) | - | 60.66 (16.81) | 61.71 (17.00) | - |  |
| Follow-up eGFR, mL/min/1.73 m^2^, mean (SD) | 72.56 (17.09) | 71.35 (18.47) | 1.21 (-0.33, 2.75) | 61.35 (18.79) | 61.49 (18.64) | -0.13 (-2.93, 2.67) |  |
| eGFR change, mL/min/1.73 m^2^, mean (SD) | 1.27 (13.14) | 0.00 (13.55) | **1.27 (0.03, 2.52)** | 0.70 (12.57) | -0.22 (12.53) | 0.92 (-0.76, 2.60) |  |

eGFR indicates estimated glomerular filtration rate; GLP-1RA, glucagonlike peptide-1 receptor agonist; SGLT2i, sodium-glucose cotransporter 2 inhibitor.

^a^GLP-1RAs included semaglutide OW T2D, oral semaglutide, dulaglutide, exenatide OW, and tirzepatide T2D.

Bold font indicates statistical significance (*P*≤0.05).

**
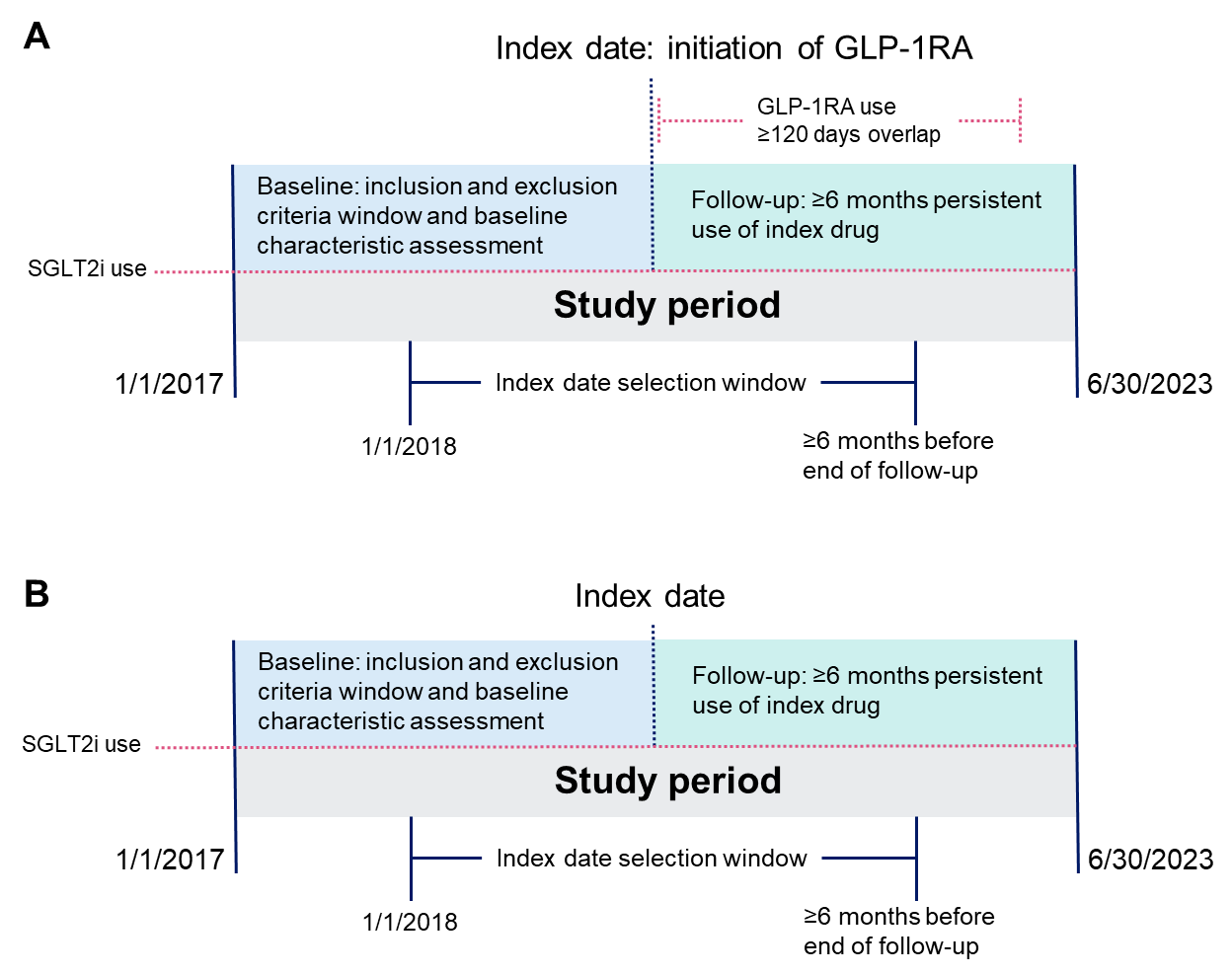
**

**Supplementary Figure 1. Study Design**
Study design for (**A**) the combination GLP-1RA and SGLT2i group and (**B**) the comparison SGLT2i group. The index date selection window was from January 1, 2018, to 180 days before the end of the study period. The baseline period was defined as the one-year period before the index date. GLP-1RA indicates glucagonlike peptide-1 receptor agonist; SGLT2i, sodium-glucose cotransporter 2 inhibitor.

**
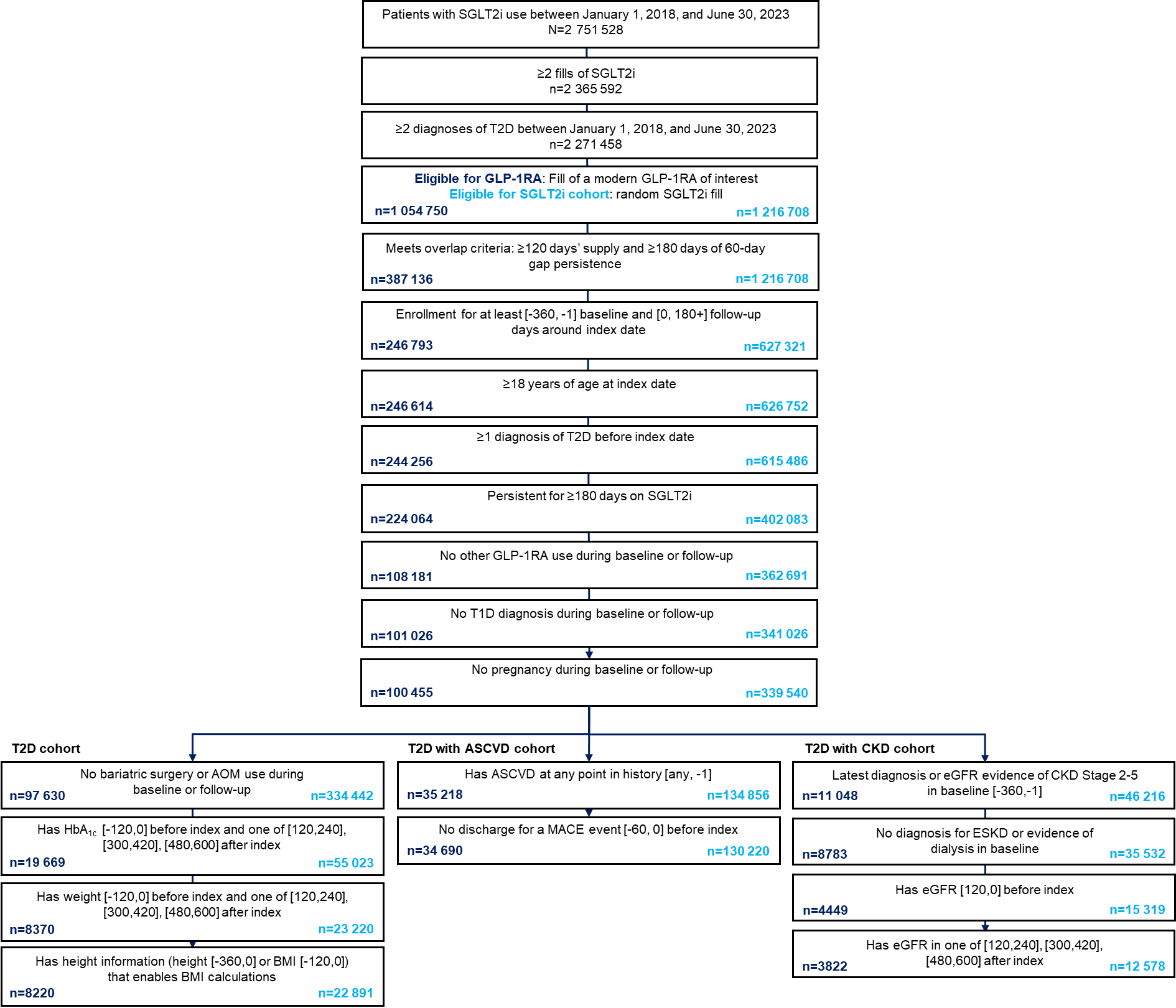
**

**Supplementary Figure 2. Patient Attrition**

AOM indicates antiobesity medication; ASCVD, atherosclerotic cardiovascular disease; BMI, body mass index; CKD, chronic kidney disease; eGFR, estimated glomerular filtration rate; ESKD, end-stage kidney disease; GLP-1RA, glucagonlike peptide-1 receptor agonist; HbA_1c_, glycated hemoglobin; MACE, major adverse cardiovascular event; SGLT2i, sodium-glucose cotransporter 2 inhibitor; T1D, type 1 diabetes; T2D, type 2 diabetes.


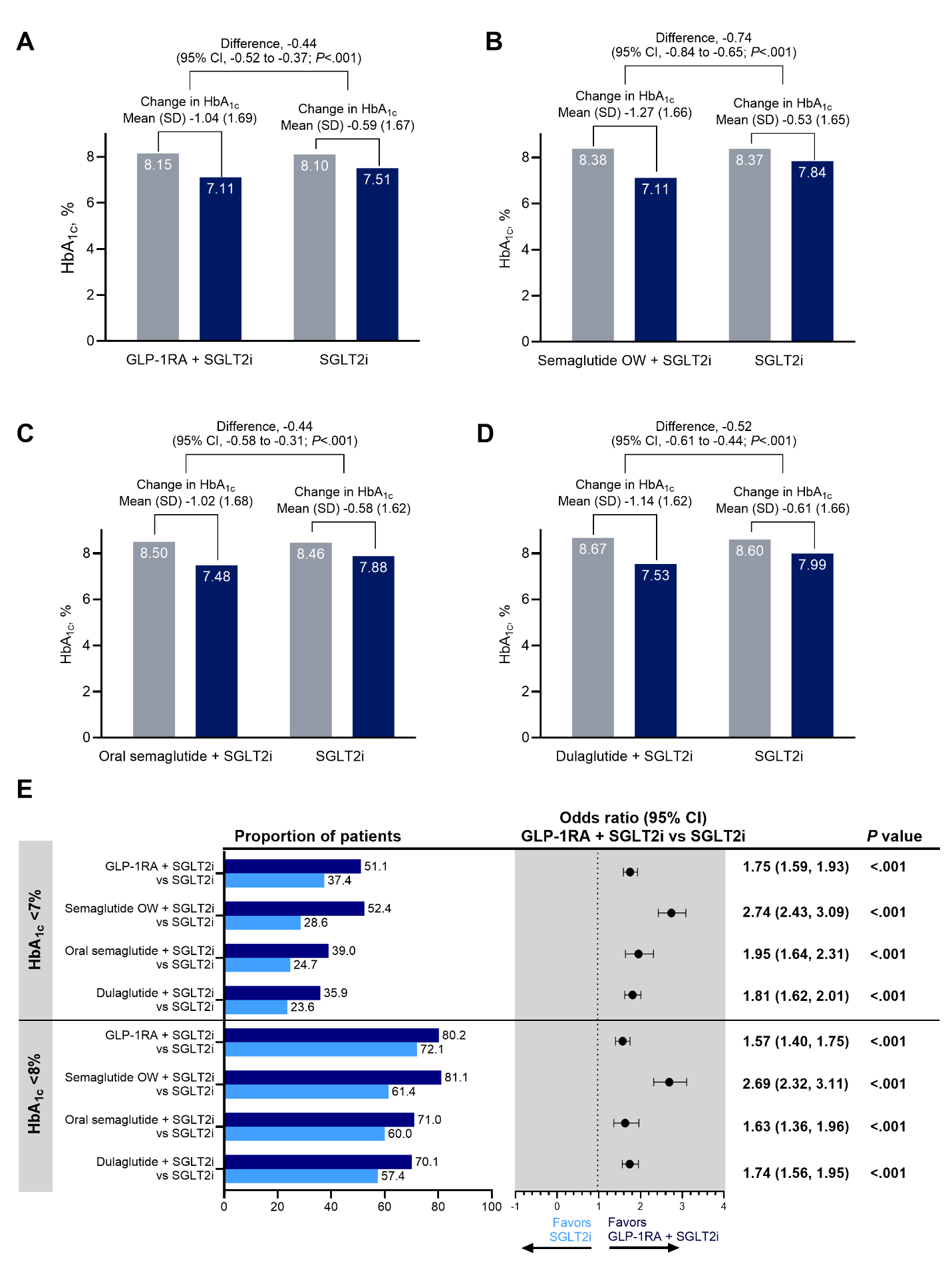


**Supplementary Figure 3. Weighted HbA_1c_ Outcomes at 12 Months for Drug Class and Individual Drug Combination Therapy With GLP-1RA and SGLT2i Compared With SGLT2i Alone**
Weighted baseline (gray bars) and 12-month follow-up HbA_1c_ levels (dark blue bars) among adults with T2D using (**A**) combination of GLP-1RA (including semaglutide OW T2D, oral semaglutide, dulaglutide, exenatide OW, and tirzepatide T2D) and SGLT2i compared with SGLT2i alone, (**B**) combination of semaglutide OW and SGLT2i compared with SGLT2i alone, (**C**) combination of oral semaglutide with SGLT2i compared with SGLT2i alone, and (**D**) combination of dulaglutide with SGLT2i compared with SGLT2i alone. (**E**) Weighted descriptive statistics and odds ratios of achieving HbA_1c_ <7% or HbA_1c_ <8% at 12 months among adults with T2D using combination GLP-1RA (including semaglutide OW T2D, oral semaglutide, dulaglutide, exenatide OW, and tirzepatide T2D) and SGLT2i therapy (dark blue bars) compared with SGLT2i alone (light blue bars). Bold odds ratios indicate statistical significance. GLP-1RA indicates glucagonlike peptide-1 receptor agonist; HbA_1c_, glycated hemoglobin; OW, once weekly; SGLT2i, sodium-glucose cotransporter 2 inhibitor; T2D, type 2 diabetes.


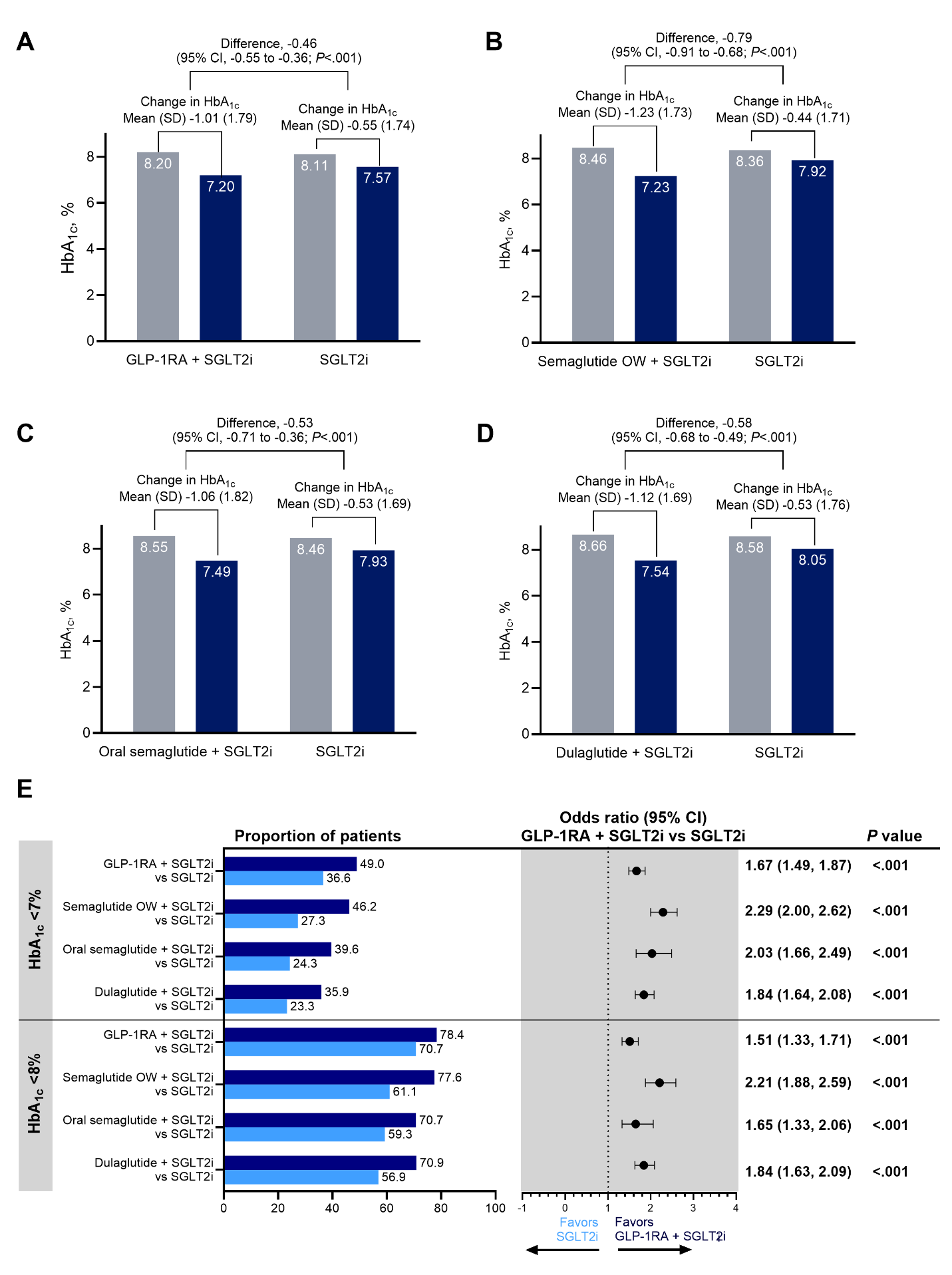


**Supplementary Figure 4. Weighted HbA_1c_ Outcomes at 18 Months for Drug Class and Individual Drug Combination Therapy With GLP-1RA and SGLT2i Compared With SGLT2i Alone**
Weighted baseline (gray bars) and 18-month follow-up HbA_1c_ levels (dark blue bars) among adults with T2D using (**A**) combination of GLP-1RA (including semaglutide OW T2D, oral semaglutide, dulaglutide, exenatide OW, and tirzepatide T2D) and SGLT2i compared with SGLT2i alone, (**B**) combination of semaglutide OW and SGLT2i compared with SGLT2i alone, (**C**) combination of oral semaglutide with SGLT2i compared with SGLT2i alone, and (**D**) combination of dulaglutide with SGLT2i compared with SGLT2i alone. (**E**) Weighted descriptive statistics and odds ratios of achieving HbA_1c_ <7% or HbA_1c_ <8% at 18 months among adults with T2D using combination GLP-1RA (including semaglutide OW T2D, oral semaglutide, dulaglutide, exenatide OW, and tirzepatide T2D) and SGLT2i therapy (dark blue bars) compared with SGLT2i alone (light blue bars). Bold odds ratios indicate statistical significance. GLP-1RA indicates glucagonlike peptide-1 receptor agonist; HbA_1c_, glycated hemoglobin; OW, once weekly; SGLT2i, sodium-glucose cotransporter 2 inhibitor; T2D, type 2 diabetes.


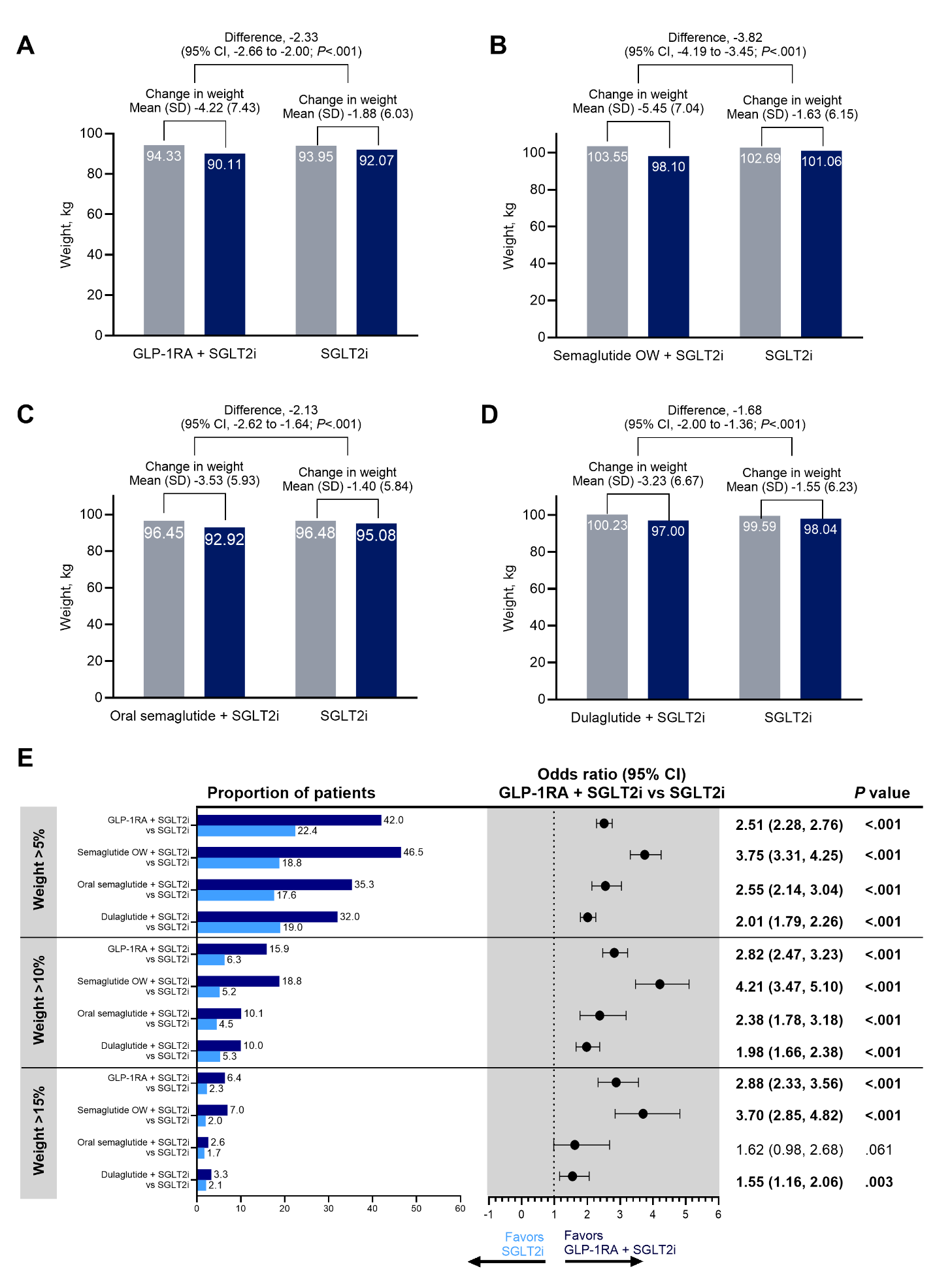


**Supplementary Figure 5. Weighted Weight Outcomes at 12 Months for Drug Class and Individual Drug Combination Therapy With GLP-1RA and SGLT2i Compared With SGLT2i Alone**
Weighted baseline (gray bars) and 12-month follow-up body weight (dark blue bars) among adults with T2D using (**A**) combination of GLP-1RA (including semaglutide OW T2D, oral semaglutide, dulaglutide, exenatide OW, and tirzepatide T2D) and SGLT2i compared with SGLT2i alone, (**B**) combination of semaglutide OW and SGLT2i compared with SGLT2i alone, (**C**) combination of oral semaglutide with SGLT2i compared with SGLT2i alone, and (**D**) combination of dulaglutide with SGLT2i compared with SGLT2i alone. (**E**) Weighted descriptive statistics and odds ratios of achieving weight loss >5%, >10%, and >15% at 12 months among adults with T2D using combination GLP-1RA (including semaglutide OW T2D, oral semaglutide, dulaglutide, exenatide OW, and tirzepatide T2D) and SGLT2i therapy (dark blue bars) compared with SGLT2i alone (light blue bars). Bold odds ratios indicate statistical significance. GLP-1RA indicates glucagonlike peptide-1 receptor agonist; OW, once-weekly; SGLT2i, sodium-glucose cotransporter 2 inhibitor; T2D, type 2 diabetes.


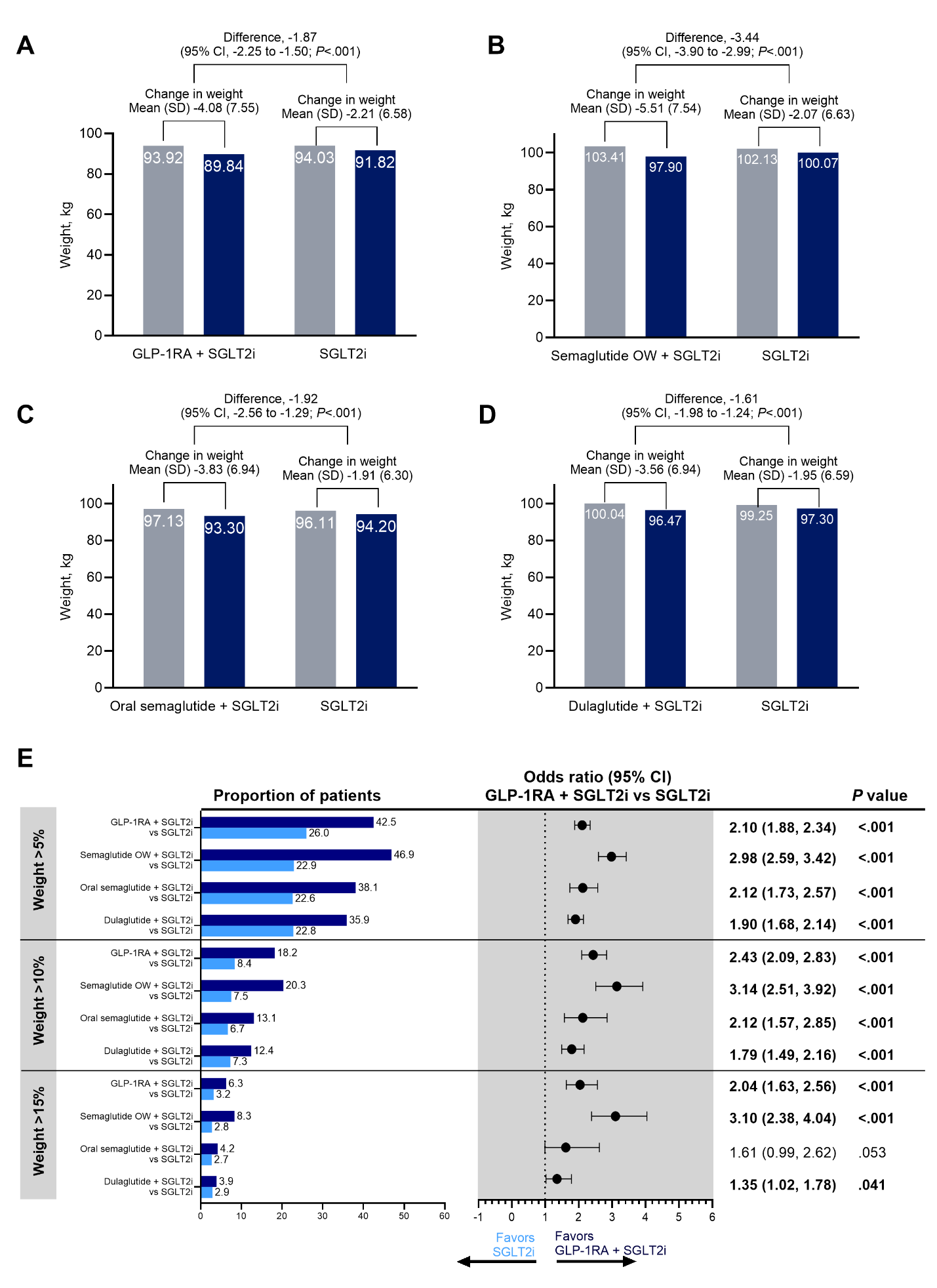


**Supplementary Figure 6. Weighted Weight Outcomes at 18 Months for Drug Class and Individual Drug Combination Therapy With GLP-1RA and SGLT2i Compared With SGLT2i Alone**
Weighted baseline (gray bars) and 18-month follow-up body weight (dark blue bars) among adults with T2D using (**A**) combination of GLP-1RA (including semaglutide OW T2D, oral semaglutide, dulaglutide, exenatide OW, and tirzepatide T2D) and SGLT2i compared with SGLT2i alone, (**B**) combination of semaglutide OW and SGLT2i compared with SGLT2i alone, (**C**) combination of oral semaglutide with SGLT2i compared with SGLT2i alone, and (**D**) combination of dulaglutide with SGLT2i compared with SGLT2i alone. (**E**) Weighted descriptive statistics and odds ratios of achieving weight loss >5%, >10%, and >15% at 18 months among adults with T2D using combination GLP-1RA (including semaglutide OW T2D, oral semaglutide, dulaglutide, exenatide OW, and tirzepatide T2D) and SGLT2i therapy (dark blue bars) compared with SGLT2i alone (light blue bars). Bold odds ratios indicate statistical significance. GLP-1RA indicates glucagonlike peptide-1 receptor agonist; OW, once weekly; SGLT2i, sodium-glucose cotransporter 2 inhibitor; T2D, type 2 diabetes.


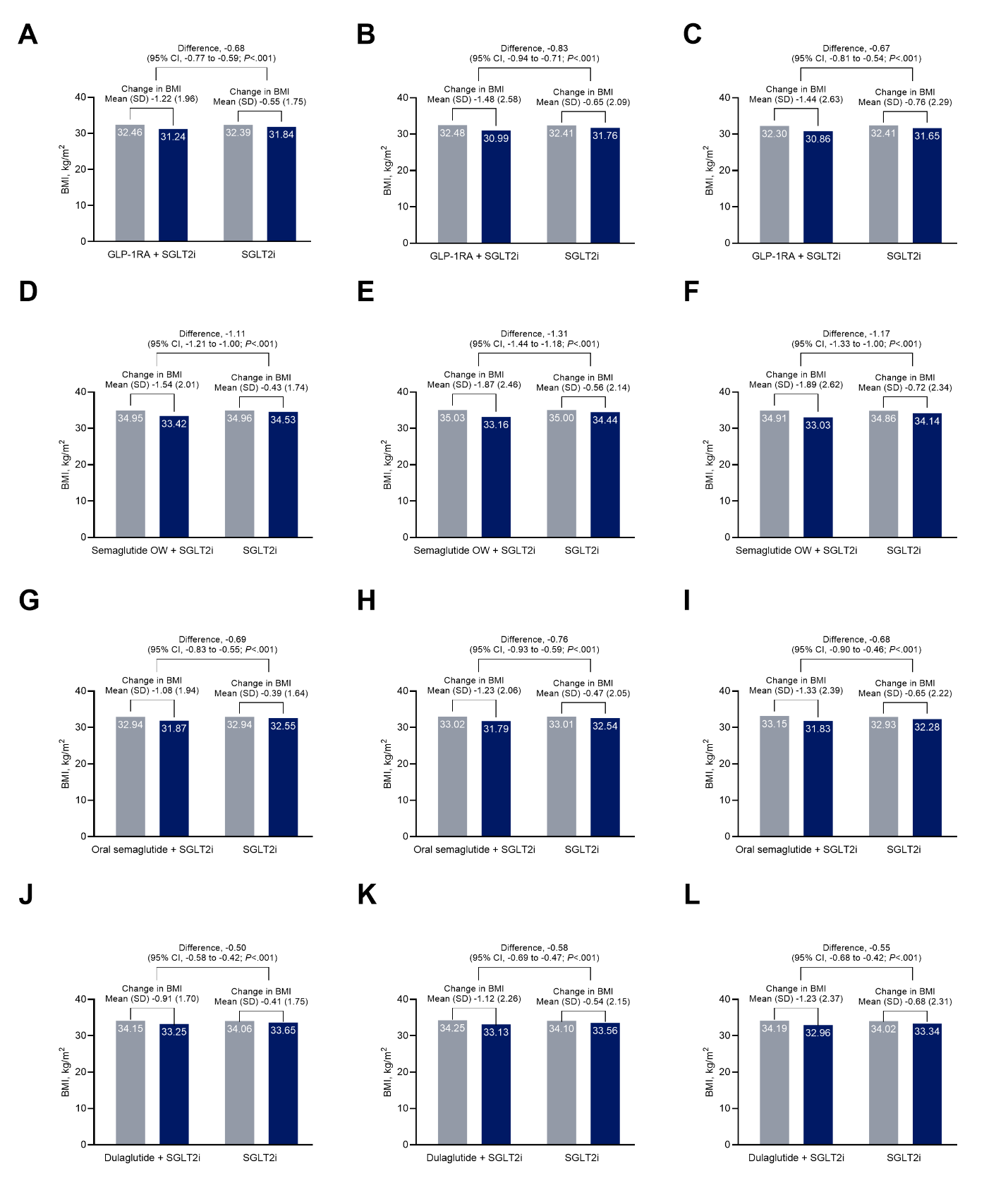


**Supplementary Figure 7. Weighted BMI Outcomes at 6, 12, and 18 Months for Drug Class and Individual Drug Combination Therapy With GLP-1RA and SGLT2i Compared With SGLT2i Alone**
Weighted baseline (gray bars) and follow-up BMI (dark blue bars) among adults with T2D using (**A-C**) combination of GLP-1RA (including semaglutide OW T2D, oral semaglutide, dulaglutide, exenatide OW, and tirzepatide T2D) and SGLT2i compared with SGLT2i alone at (**A**) 6 months, (**B**) 12 months, and (**C**) 18 months; (**D-F**) combination of semaglutide OW and SGLT2i compared with SGLT2i alone at (**D**) 6 months, (**E**) 12 months, and (**F**) 18 months; (**G-I**) combination of oral semaglutide with SGLT2i compared with SGLT2i alone at (**G**) 6 months, (**H**) 12 months, and (**I**) 18 months; and (**J-L**) combination of dulaglutide with SGLT2i compared with SGLT2i alone at (**J**) 6 months, (**K**) 12 months, and (**L**) 18 months. BMI indicates body mass index; GLP-1RA, glucagonlike peptide-1 receptor agonist; OW, once weekly; SGLT2i, sodium-glucose cotransporter 2 inhibitor; T2D, type 2 diabetes.
